# A Comparison Between High- and Low-Performing Lambs and Their Impact on the Meat Quality and Development Level Using a Multi-Omics Analysis of Rumen Microbe–Muscle–Liver Interactions

**DOI:** 10.3390/microorganisms13040943

**Published:** 2025-04-19

**Authors:** Haibo Wang, Jinshun Zhan, Shengguo Zhao, Haoyun Jiang, Haobin Jia, Yue Pan, Xiaojun Zhong, Junhong Huo

**Affiliations:** 1 Jiangxi Province Key Laboratory of Animal Green and Healthy Breeding, Institute of Animal Husbandry and Veterinary, Jiangxi Academy of Agricultural Science, Nanchang 330200, China; wanghaibo8815@163.com (H.W.); zhanjinshun1985@163.com (J.Z.); jianghaoyun1995@163.com (H.J.); jiahaobin@jxaas.cn (H.J.); py13782525871@163.com (Y.P.); zhongcaoyangchu@163.com (X.Z.); 2 College of Animal Science and Technology, Gansu Agricultural University, Lanzhou 730070, China; zhaosg@gsau.edu.cn; 3 Provincial Development and Research Institute of Ruminants in Gansu, Lanzhou 730070, China

**Keywords:** body weight, Hu lamb, microbe–muscle–liver axis, rumen function, slaughter performance

## Abstract

Through an integrated multi-omics analysis of rumen microbial communities, muscle transcriptomes, metabolic profiles, and liver metabolic profiles, this study systematically compared high- and low-performing lambs to elucidate their divergent effects on meat quality attributes and growth development. A total of 100 male lambs with similar birth weight (3.07 ± 0.06 kg) were selected within 72 h. All test lambs were synchronized weaning at 45 days of age and uniformly fed the same diet (total mixed ration) in the same pen until 180 days of age, with ad libitum access to food and water throughout this period. Subsequently, the eight lambs with the highest (HADG) and lowest (LADG) average daily gains were slaughtered for performance evaluation and multi-omics analysis. This study found that HADG lambs increased body weight, muscle fiber diameter, eye muscle area, improved amino acid (histidine, arginine, valine, isoleucine, essential amino acid/total amino acid, and essential amino acid/nonessential amino acid), and fatty acid (linoleic acid, behenic acid, and arachidonic acid) composition enhanced rumen enzymes (pepsase, lipase, xylanase, amylase, and carboxymethyl cellulose) and promoted efficient fermentation (*p* < 0.05). Analysis of microbial populations indicated a notable increase in *Prevotella* levels within the rumen of HADG lambs. Furthermore, the rumen markers *Schwartzia* and *Streptococcus* exhibited significant correlations with differential meat quality traits. Analysis of the muscle transcriptome indicated a significant correlation between the turquoise module and host phenotypes, particularly body weight. Additionally, muscle metabolism is primarily concentrated within the black module; however, it exhibits a significant correlation with the host body phenotype in the yellow module (*p* < 0.05). Moreover, liver metabolites, rumen microbes, host phenotype, and muscle transcripts were significantly correlated (*p* < 0.05). In conclusion, the interactions among rumen microbes, muscle, and liver in lambs promote rumen fermentation, which in turn regulate muscle transcriptional activity and modify metabolic profiles in both the liver and muscle. Moreover, *PCK1*, *SPP1*, *FGF7*, *NR4A1*, *DUSP5*, *GADD45B*, etc., can be candidate genes for muscle growth and development. This finding provides a theoretical basis for further exploiting the production potential of Hu lambs.

## 1. Introduction

Sheep (*Ovis aries*) rely on their gastrointestinal microbiota to metabolize plant-based feeds, including straw, hay, silage, and grass, into products such as meat, wool, fur, and milk [1], and their metabolic transformation is intricately associated with the microbial composition of the host’s gastrointestinal tract [2,3]. Research has shown that rumen microorganisms play a crucial role in influencing host phenotypes, including metabolic processes in muscle [4,5,6], body weight [7], feed efficiency [8,9], skeletal muscle development, and meat quality [6,10,11]. Nevertheless, the composition of gastrointestinal microbiota in animals is influenced by a variety of factors, such as the host’s genetic background, age, sex, dietary habits, and geographic location [7,12,13]. Enhancing feed efficiency in sheep serves as a crucial indicator within the sheep production process and significantly contributes to the improvement of economic efficiency. Furthermore, sheep body weight is a key growth indicator influenced by genetics, environment, nutrition, and gut microbiota [7,14,15,16], and it can influence meat production and reproductive performance [17,18]. The microbial fermentation occurring in the rumen of ruminants produces volatile fatty acids (VFAs), which are crucial for the physiological functions of the host. These VFAs account for approximately 70% of the energy requirements of ruminants and are integral to the regulation of bile acid metabolism, the functioning of the liver and pancreas, the maintenance of intestinal barrier integrity, and the overall health of the host [7,19,20,21]. Research demonstrates that small molecule compounds, including VFAs, bile acids, succinic acid, and betaine, play a significant role in modulating key signaling molecules that are integral to animal growth and development via interactions between microbes and their hosts [7,21,22,23,24,25,26]. Additionally, variations in the molar content and ratios of different VFAs are crucial for targeting and regulating host metabolism [20,21].

Skeletal muscle is a significant metabolic organ that can affect the metabolism of other tissues and organs [27], including adipose tissue [28], the nervous system [29], and liver metabolism [30]. Moreover, metabolic homeostasis is influenced by various cell types in the body, facilitating interactions among different organs and tissues [27], such as muscle [31], adipose tissue [28], the liver [30], and the nervous system [29]. Skeletal muscle plays a role in regulating liver metabolic functions, while liver-derived metabolic factors are essential for interactions between the liver, other organs, and gastrointestinal microbes [22,30,32]. Additionally, interactions between microbes and the host are mediated by metabolites, such as VFAs and bile acids, which serve as signaling molecules recognized by host receptors to influence metabolic pathways associated with energy metabolism and food consumption [33]. In addition, the live weight and growth rate of lambs are important economic traits, and studies have demonstrated that animals with elevated Kleiber ratios (growth rate/body weight^0.75^) are considered effective feed users [34,35]. Thus, we hypothesized that interactions between the rumen–muscle–liver are influenced by variations in the molar content and proportion of VFAs in the rumen. These alterations are thought to be instrumental in the regulation of metabolic homeostasis within muscle and liver tissues, which subsequently influence both the growth and development of lambs, as well as the quality of the meat produced. Therefore, this study used a comparison between high- and low-performing lamb and their impact on the meat quality and development level using a multi-omics the analysis of rumen–muscle–liver interactions, which could provide a theoretical basis for improving meat quality and growth in lambs.

## 2. Materials and Methods

### 2.1. Lamb and Experimental Design

One hundred Hu lambs (males) of similar birth weight (3.07 ± 0.06 kg) and age (within 72 h of life) were selected from the Jiangxi Academy of Agricultural Sciences Science and Technology Service Workstation, Ganzhou Lvlinwan Agricultural and Animal Husbandry Co., Ltd. (Ganzhou, China), that were synchronized weaning at 45 days of age and reared until 180 days of age. During the experimental period, 100 lambs were fed under the same feeding conditions and growing environment (same pen, half open-front livestock house, and natural lighting) and fed the company’s total mixed ration at 08:30 and 17:30 daily. At 180 days of age, based on the Kleiber ratio (growth rate/body weight^0.75^) [34], we selected the Hu lamb with the highest (HADG, *n* = 8) and lowest (LADG, *n* = 8) average daily gain from 100 lambs for evaluation of relevant parameters, respectively. All experimental procedures were approved (10 May 2022) by the Institutional Animal Care and Use Committee of Jiangxi Academy of Agricultural Sciences (2010–JAAS–XM–01). Slaughter procedures were performed according to operating procedures of livestock and poultry slaughtering for sheep and goats (NY/T 3469–2019, Operating procedures of livestock and poultry slaughtering sheep and goat, Ministry of Agriculture, Beijing, China, 2019) [36]. Lamb weights were measured using calibrated electronic scales.

### 2.2. Sample Collection and Processing

Rumen fluid was stored by liquid nitrogen flash freezing at −80 °C for VFAs, enzyme activity, and microbial sequencing. Rumen and longissimus dorsi tissues were collected for morphological analysis. Furthermore, longissimus dorsi and liver samples were collected and frozen in liquid nitrogen and sent to the laboratory for storage at −80 °C for multi-omics assays and the determination of amino acids and fatty acids. Additionally, longissimus dorsi was collected for routine nutrient and meat physical traits analysis.

### 2.3. Analyzing Slaughtering Performance and Meat Quality Characteristics

The thickness of the adipose tissue between the 12th and 13th ribs (referred to as back fat thickness) and the thickness of the tissue 11 cm from the midline of the back (designated as rib thickness) were measured using vernier calipers (Mitutoyo, Kawasaki, Japan). The length and width of the longissimus dorsi cross section between the 12th and 13th ribs were measured using vernier calipers (Mitutoyo, Kawasaki, Japan), and the eye muscle area was calculated (length × width × 0.7) [37]. Furthermore, the measurement of a*, b*, and L* values of longissimus dorsi was conducted using a colorimeter (CR–10; Minolta, Japan). The pH of the longissimus dorsi was evaluated utilizing a calibrated (standards of 4.00 and 6.86) pH meter (Testo 205; Testo AG, Germany), which involved inserting the pH electrode into the fresh meat sample, ensuring that the electrode’s tip made complete contact with the specimen. Furthermore, shear forc, water loss rate (about 5 g of meat on the top and bottom of each pad 18 layers of filter paper, and weighed after being pressed by a 35 kg square iron for 5 min, and the value of the difference between the pre-pressing and post-pressing meat weights/pre-pressing meat weight × 100 was calculate), cooking loss (A 100 g sample of meat was boiled in water for 45 min, cooled at room temperature for 30 min, and then weighed, using the weight of meat after cooking/meat before cooking × 100), crude protein moisture, crude fat, and amino acids of the longissimus dorsi were determined with reference to the detailed description of Zhan et al. [37]. In addition, longissimus dorsi fatty acids were determined according to the method described by Wang et al. [38].

### 2.4. Morphometric Analysis of the Longissimus Dorsi and Rumen Tissue

The rumen (abdominal sac tissue) and muscle tissues (longissimus dorsi between the 12th and 13th ribs) were collected and fixed in 4% paraformaldehyde. After tissue fixation, the tissues were sent to Wuhan Service Biotechnology Co., Ltd. (Wuhan, China) to be dehydration and wax leaching (75% alcohol for 4 h, 85% alcohol for 2 h, 90% alcohol for 2 h, 95% alcohol for 1 h, anhydrous ethanol I for 30 min, anhydrous ethanol II for 30 min, alcohol benzene for 8 min, xylene II for 8 min, 65 °C melting paraffin I for 1 h, 65 °C melting paraffin II for 1 h, 65 °C melting paraffin III for 1 h), embedding, sectioned, hematoxylin staining (4 min), eosin staining (95% alcohol was dehydrated for 1 min and eosin was dyed for 15 s), dehydration and sealing (absolute ethanol I for 2 min, absolute ethanol II for 2 min, absolute ethanol III for 2 min, normal butanol I for 2 min, normal butanol II for 2 min, xylene I for 2 min, xylene II for 2 min, sealing with neutral gum), microscope inspection, and image acquisition and analysis. Finally, the sections were examined using an Eclipse Ci–L photomicroscope (Nikon, Shinagawa, Japan), and the morphological characteristics of muscle fibers (muscle fiber diameter (MFD), number of muscle fibers (NMF), density of muscle fibers (DMF)) and rumen morphology (papilla height, papilla width, muscle layer, stratum corneum, basal layer thickness, stratum granular, and stratum spinosum) were analyzed using Image–Pro Plus 6.0 image processing software (Media Cybernetics, Inc., Rockville, MD, USA).

### 2.5. Analysis of Rumen VFAs and Digestive Enzymes

The molar concentrations of VFAs in the rumen were measured using gas chromatography (GC-7890B, Agilent Technologies, Petaling Jaya, Malaysia) with an internal standard method involving 2–ethylbutyric acid, following the procedures outlined by Wang et al. [39]. Subsequently, molar ratios of VFAs were calculated (molar content of each VFA/TVFA × 100). Furthermore, lipase (FK7100117–A), beta glucosidase (GLU, FK7100195–A), xylanase (FK7100126–A), amylase (FK7100105–A), microcrystalline cellulose (MCC, FK7100202–A), and carboxymethyl cellulose (CMC, FK7100120–A) were measured in rumen fluid according to the specific guidelines provided in the kit (Shanghai Kexing trading Co., Ltd., Shanghai, China).

### 2.6. DNA Extraction and Analysis of Bacterial Community in Rumen

The bacterial DNA was extracted from 16 rumen fluid samples using the TGuide S96 Magnetic Stool DNA Kit (Tiangen Biotech (Beijing) Co., Ltd., Beijing, China). Initially, a specific proportion of quantitative reference sequences (spike-in DNA) was added to the sample DNA, followed by PCR amplification with designed conserved primers (338F: 5′–ACTCCTACGGGAGGCAGCA–3′ and 806R: 5′–GGACTACHVGGGTWTCTAAT–3′.). Next, the products were then purified, quantified, and homogenized to create sequencing libraries and ensure quality control. Subsequently, the quality-checked libraries were sequenced using Illumina Novaseq 6000 (Beijing Biomarker Technologies Co., Ltd., Beijing, China) to obtain raw data. Moreover, the raw data were executed according to standardized procedures for filtering, removing primer sequence, double-ended reads for splicing, removing chimeras and internal reference sequence, thereby obtaining high-quality sequences for subsequent analysis. Finally, high-quality data were denoised with the DADA2 [40] method in QIIME2 (version 2020.6), and ASVs were classified using the Naive Bayes classifier based on the SILVA database (Release138, https://www.arb-silva.de/, accessed on 4 April 2024), with a 70% confidence threshold.

### 2.7. Transcriptome Sequencing and Bioinformatics Analysis

The total RNA was extracted from the longissimus dorsi of Hu lambs using a Trizol kit (Invitrogen, Carlsbad, CA, USA) according to the instructions of the kit. RNA extraction was carried out using the NanoDrop2000 (Thermo Scientific, Waltham, MA, USA) and Agilent 2100 (Agilent, Santa Clara, CA, USA) after evaluation and qualification. Extracting the RNA that meets the standard was enriched through the application of mRNA Capture Beads. Following the purification of the beads, the mRNA was subjected to fragmentation via high-temperature treatment. Subsequently, the fragmented mRNA served as a template for the synthesis of the first strand of cDNA within a reverse transcriptase reaction system. While synthesizing the second strand of cDNA, end repair and A-tailing are completed. Subsequently, adapters are ligated, and the target fragments were selected through purification utilizing Hieff NGS^®^ DNA Selection Beads. Following this, PCR library amplification was conducted, and ultimately, detection was performed using the Illumina Novaseq X Plus platform (Gene Denovo Biotechnology Co., Guangzhou, China). Then, the raw reads are filtered using fastp (version 0.18.0) [41] to obtain clean reads. Finally, the paired-end clean reads were localized to the reference genome (Ensemble_release101) by using HISAT2 2.1.0 [42], while the mapped reads of each sample were assembled by using StringTie v1.3.1 [43,44] in a reference-based approach, and FPKM (transcribed fragments per kilobase per million mapped reads) values were calculated to quantify their expression, with fold change ≥ 2 and FDR < 0.05 as screening differential genes. Finally, the identified genes were annotated using the Kyoto Encyclopedia of Genes and Genomes (KEGG) [45] (https://www.genome.jp/kegg/, accessed on 9 June 2023) and the annotated genus were then mapped to the KEGG items.

### 2.8. Metabolome Sequencing and Bioinformatics Analysis

Briefly, 50 mg of longissimus dorsi sample was transferred to an EP tube. After the addition, extract solution containing isotopically labeled internal standard mixture vortexed for 30 s, ice water bath sonicated (10 min), incubated precipitate proteins (1 h, −40 °C), and centrifuged (12 000 rpm, 4 °C, 15 min). Chromatographic separation of the target compounds was carried out on a Waters ACQUITY UPLC BEH Amide column using a Vanquish ultra-high performance liquid chromatograph (Thermo Fisher Scientific), and the mass spectrometer was capable of data acquisition by Gene Denovo Biotechnology Co. (Guangzhou, China) under the control of the Xcalibur (version 4.4) control software. Additionally, liver metabolomic data refer to the detailed description in the previous publication by Wang et al. [22]. Finally, the identified metabolites were annotated using the KEGG [45] (https://www.genome.jp/kegg, accessed on 12 June 2023) database, and the annotated metabolites were then mapped to the KEGG Pathway database, with variable importance for the projection (VIP) ≥ 1 and *p* < 0.05 as screening differential genes.

### 2.9. Data Statistics and Analysis

The carcass, longissimus dorsi physical traits, longissimus dorsi base nutritional components, rumen VFAs, digestive enzymes, and morphology by a normality test was performed using SPSS software (version 26.0, SPSS Inc., Chicago, IL, USA), followed by an independent samples t-test for data analysis. The data were represented as the means ± Standard Error. *p* < 0.05 was considered significant. The microbiota alpha diversity index of rumen fluid samples was assessed with QIIME2. The beta diversity of rumen microbiota communities was analyzed using binary Jaccard indices with principal coordinates analysis (PCoA) and non-metric multi-dimensional scaling (NMDS). Linear discriminant analysis (LDA = 2.5) effect size (LEfSe) was used to find statistically different biomarkers. The rumen microbial top 80 correlation network graph was constructed based on a correlation coefficient of R > 0.3 and a *p* < 0.05. Next, OPLS-DA (version 1.6.2) was employed to identify metabolic differences between the HADG and LADG groups. Longissimus dorsi transcriptome and metabolome modules were enriched according to power = 8, maximum number of modules 20, and minimum number of metabolites/genes in a module 50. The top 30 frequency liver metabolite module/rumen microbe correlations were retained in accordance with the data containing at least one set of correlation coefficients with absolute values within the top 30, which were used for correlation chord plots (Retain *p*-values containing with at least one correlation set where CCP < 0.05) and relevance network diagram (|CC| > 0.8 and CCP < 0.05).

## 3. Results

### 3.1. Analysis of Carcass and Meat Physical Traits in Lambs of Different Growth and Development

Table 1 shows that HDAG lambs exhibited significantly higher body weight, eye muscle area, and muscle fiber diameter compared to LADG lambs (*p* < 0.05). However, HDAG lambs also show a significantly lower number of muscle fibers and density of muscle fibers (*p* < 0.05), with no significant effect on back fat thickness, rib thickness, meat color (a*_45 min_, b*_45 min_, and L*_45 min_), pH_45 min_, shear force, water loss rate, and cooking loss (*p* > 0.05).

### 3.2. Analysis of the Nutritional Components in Lambs of Different Growth and Development

Table 2 indicates that histidine (His), arginine (Arg), valine (Val), isoleucine (Ile), EAAs/TAA, EAAs/NEAA, linoleic acid (C18:2n6c), behenic acid (C22:0), and arachidonic acid (C20:4n6) were significantly higher in HDAG lambs than in LADG (*p* < 0.05), but significantly lower number of methionine (Met) and myristic acid (*p* < 0.05), with no significant effect on crude protein, moisture, and crude fat (*p* > 0.05).

### 3.3. Analysis of Rumen Fermentation Parameters and Histomorphology in Lambs

#### 3.3.1. Analysis of the Rumen VFAs in Lambs of Different Growth and Development

Table 3 rumen VFA results show that HDAG lambs had significantly higher levels of AA, PA, IBA, BA, VA, TVFA, and PAR compared to LADG lambs (*p* < 0.05). Conversely, the AA:PA, AAR, and IVAR were significantly lower in HDAG lambs (*p* < 0.05). There were no significant differences observed in IVA, IBAR, BAR, and VAR between the two groups (*p* > 0.05).

#### 3.3.2. Analysis of the Rumen Digestive Enzyme and Histomorphology in Lambs

In Table 4, the digestive enzyme result indicates that pepsase, lipase, xylanase, amylase, and CMC were significantly higher in HDAG lambs than in LADG (*p* < 0.05), but with no significant effect on GLU and MCC (*p* > 0.05). Finally, in Table 4, the histomorphology indicates that papilla height, papilla width, muscle layer, stratum corneum, basal layer thickness, stratum granular, and stratum spinosum was not significant in HDAG lambs and in LADG (*p* > 0.05).

### 3.4. Analysis of the Rumen Microbiota of HADG and LADG Lambs

#### 3.4.1. Analysis of the Microbiota Diversity of the Rumen

**Figure 1:**
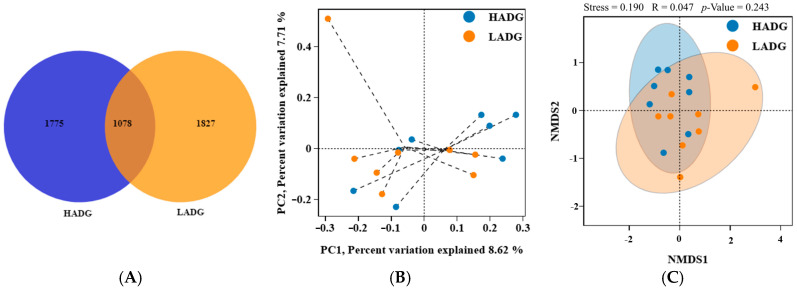
Analysis of the rumen microbiota diversity of the HADG and LADG lambs. ASV Venn diagram analysis (**A**) of HADG and LADG lambs. PCoA analysis (**B**) and NMDS analysis (**C**) of HADG and LADG lambs.

Table 5 shows that rumen microbial α–diversity (ACE, Chao1, Shannon, and Simpson) was not significantly different between LADG and HADG lambs (*p* > 0.05). A subsequent analysis of these sequences utilizing Venn diagrams indicated that a total of 4680 ASVs were identified, with 1078 ASVs being common to two distinct groups (Figure 1A). Furthermore, PCoA (Figure 1B) and NMDS (Figure 1C) results based on the binary Jaccard method showed that rumen microbial β–diversity was also not significantly different between LADG and HADG lambs (*p* > 0.05).

#### 3.4.2. Analysis of the Rumen Microbiota Composition of the Rumen

**Figure 2:**
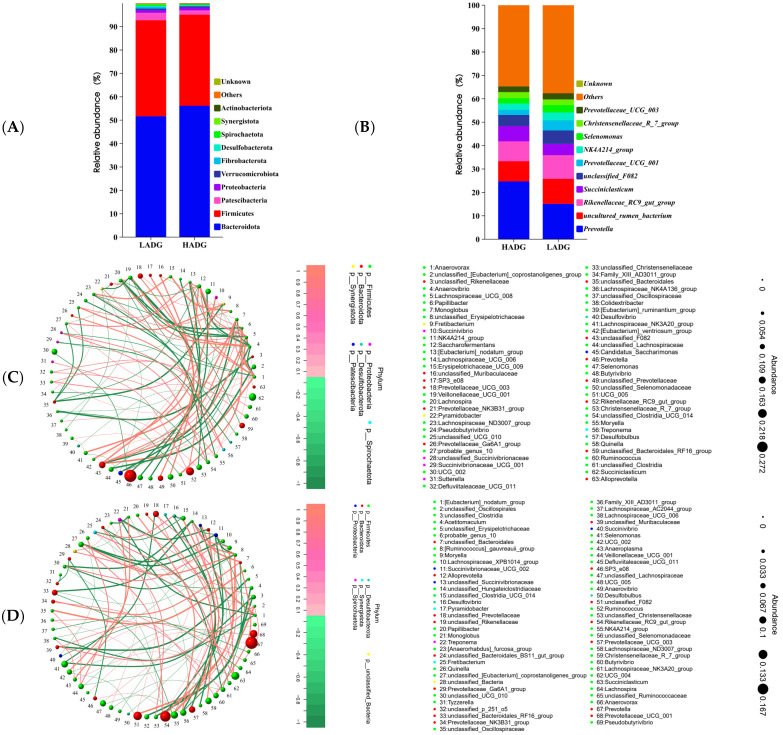
Analysis of rumen microbial composition of HADG and LADG lambs. HADG and LADG lamb rumen microbiota phylum (**A**) and genus (**B**) level top 10 stacking diagrams. Network diagrams illustrating the microbiota of the lamb rumen for HADG (**C**) and LADG (**D**) have been constructed, and the network diagrams showed the presence of significantly associated microbiota in top 80 (*p* < 0.05).

The analysis of rumen microbiota revealed that the dominant phylum were *Firmicutes* and *Bacteroidetes*, which together comprised over 92.87% of the total bacterial population in the community (Figure 2A). At the phylum level, there were no overall significant differences in rumen microbes (top 10) among the different growth and developmental lambs, however only *Desulfobacterota* being significantly higher in LADG than in HADG group lambs (*p* < 0.05) (Figure 2A). At the genus level, the top 10 abundance accounted for more than 62.39% (Figure 2B). Meanwhile, compared with the LADG group, the relative abundance of *Prevotella* in the HADG group has been significantly increased (*p* < 0.05), while the relative abundance of *uncultured_rumen_bacterium*, *Succiniclasticum*, *Prevotellaceae_UCG_001*, *Christensenellaceae_R_7_group*, *Rikenellaceae_RC9_gut_group*, *unclassified_F082*, *NK4A214_group*, *Selenomonas*, and *Prevotellaceae_UCG_003* between HADG and LADG lambs (*p* > 0.05) (Figure 2B). Moreover, the construction of top 80 microbiota network diagrams at the genus level for the rumen of lambs in groups LADG and HADG revealed a significant correlation among the microbiota present (Figure 2C,D). Notably, both groups were predominantly characterized by *Firmicutes* (Figure 2C,D).

#### 3.4.3. Analysis of the Rumen Microbiome in Correlation with Host Phenotype

**Figure 3:**
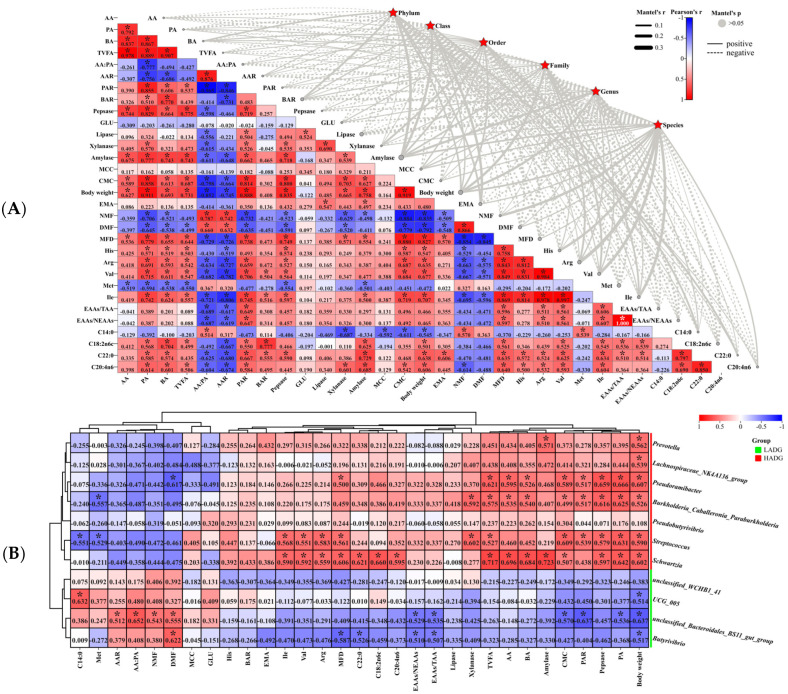
Analysis of rumen microbial–host phenotype correlation in HADG and LADG lambs. (**A**) Rumen microbial–host phenotype correlation in HADG and LADG lambs. The width of the edges is representative of Mantel’s r statistics associated with the respective distance correlations, while the color of the edges reflects the significance of Mantel’s *p* statistic, where a gray line denotes indicating *p* > 0.05. (**B**) Heat map analysis of rumen genus level microbiota marker correlation with phenotype of HADG and LADG lambs. Note: the values in the correlation heat map indicate the correlation coefficients. * In the correlation heat map, we indicate *p* < 0.05. Abbreviations: HADG: highest average daily gain; LADG: lowest average daily gain.

Overall, there was no significant correlation between rumen microbiota and rumen VFAs, digestive enzymes, carcasses, and meat quality characteristics in lambs (Figure 3A). Furthermore, LEfSe (LDA = 2.5) analysis of lamb rumen biomarkers identified 20 significant biomarkers, with 8 linked to LADG and 12 to HADG (Supplementary Figure S1). Moreover, 4 biomarkers (*Butyrivibrio*, *UCG_005*, *unclassified_Bacteroidales_BS11_gut_group*, and *unclassified_WCHB1_41*) were found at the genus level in LADG lambs, whereas 7 biomarkers (*Pseudobutyrivibrio*, *Schwartzia*, *Lachnospiraceae_NK4A136_group*, *Burkholderia_Caballeronia_Paraburkholderia*, *Pseudoramibacter*, *Streptococcus*, and *Prevotella*) were found at the genus level in HADG lambs (Supplementary Figure S1). However, the correlation between rumen differential markers and host phenotypes was below 0.8 (Figure 3B). In HADG lambs, rumen differential markers (*Pseudobutyrivibrio*, *Schwartzia*, *Lachnospiraceae_NK4A136_group*, *Burkholderia_Caballeronia_Paraburkholderia*, *Pseudoramibacter*, *Streptococcus*, and *Prevotella*) were positively correlated with significantly higher phenotypes (AA, PA, BA, TVFA, PAR, pepsase, lipase, xylanase, amylase, CMC, body weight, EMA, MFD, His, Arg, Val, Ile, EAAs/TAA, EAAs/NEAAs, C18:2n6c, C22:0, and C20:4n6), and negatively correlated with significantly lower phenotypes (C14:0, Met, AA:PA, AAR, NMF, and DMF) in lambs (Figure 3B). However, it is worth noting that *Schwarzia* and lipase, *Streptococcus* and EMA; *Pseudobutyrivibrio* and C22:0, *Lachnospiraceae_NK4A136_group* and muscles (His, Arg, Val, Ile, EAAs/TAA, EAAs/NEAAs), and *Pseudobutyrivibrio* and *Prevotella* and muscle (EAAs/TAA, EAAs/NEAAs) were negatively correlated (Figure 3B). Moreover, *Schwartzia* and *Streptococcus* were significantly positively correlated with the meat traits Arg, Val, Ile, and MFD, and *Schwartzia* was significantly positively correlated with fatty acids (C18:2n6c, C22:0, and C20:4n6) (*p* < 0.05) (Figure 3B). In parallel, *Schwartzia*, *Burkholderia_Caballeronia_Paraburkholderia*, and *Pseudoramibacter* was significantly positively correlated with VFAs (AA, PA, BA, and TVFAs) (*p* < 0.05) (Figure 3B). Notably, body weight was significantly positively correlated with AA, PA, BA, TVFA, PAR, Pepsase, Xylanase, Amylase, CMC, MFD, His, Arg, Val, Ile, C18:2n6c, C22:0, C20:4n6, *Schwartzia*, *Lachnospiraceae_NK4A136_group*, *Burkholderia_Caballeronia_Paraburkholderia*, *Pseudoramibacter*, *Streptococcus*, and *Prevotella*, but it was significantly negatively correlated with AA:PA, AAR, DMF, NMF, C14:0, *Butyrivibrio*, *UCG_005*, and *unclassified_Bacteroidales_BS11_gut_group* (*p* < 0.05) (Figure 3A,B).

### 3.5. Analysis of Transcriptome Profiling of Longissimus Dorsi

#### 3.5.1. Analysis of Transcriptome Differences in the Longissimus Dorsi of Lambs

**Figure 4:**
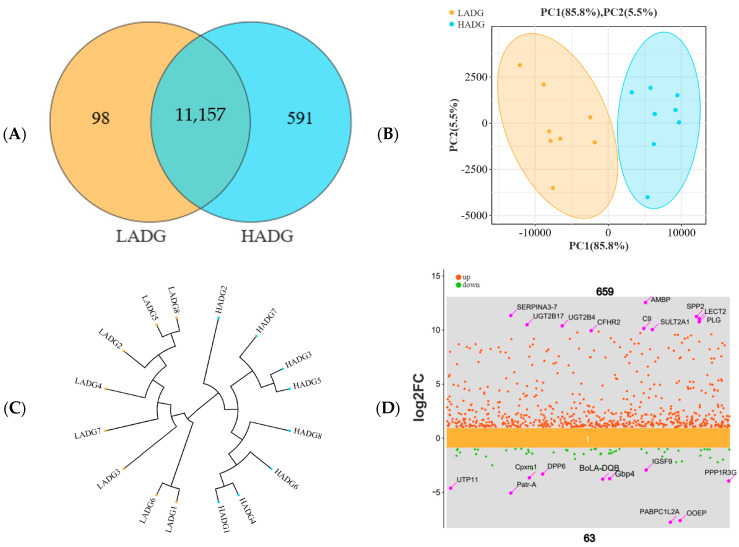
Analysis of transcriptome differences in the longissimus dorsi of the HADG and LADG lambs. Venn diagram (**A**), PCA (**B**), cluster dendrogram (**C**), and differential gene scatterplot (**D**) between HADG and LADG lambs. Abbreviations: HADG: highest average daily gain; LADG: lowest average daily gain.

In transcriptome sequencing, a Venn diagram revealed that these sequences were assigned to 11,846 genes, of which 11,157 genes were shared between HADG and LADG group lambs (Figure 4A). Additionally, the PCA sample is illustrated in Figure 4B, along with the sample cluster dendrogram presented in Figure 4C, revealing a clear separation between the HADG and LADG group. Meanwhile, scatter plots by multiplicity of difference > 2 and significance threshold FDR value < 0.05 revealed that 659 genes were up-regulated and 63 genes were down-regulated in the HADG compared to the LADG group lambs (Figure 4D). Furthermore, the longissimus dorsi discrepancy gene and body weight are strongly correlated, especially *SPP1* (R = 0.984) (Supplementary Table S1).

#### 3.5.2. Host Phenotype and Longissimus Dorsi Gene Module Association Analysis

**Figure 5:**
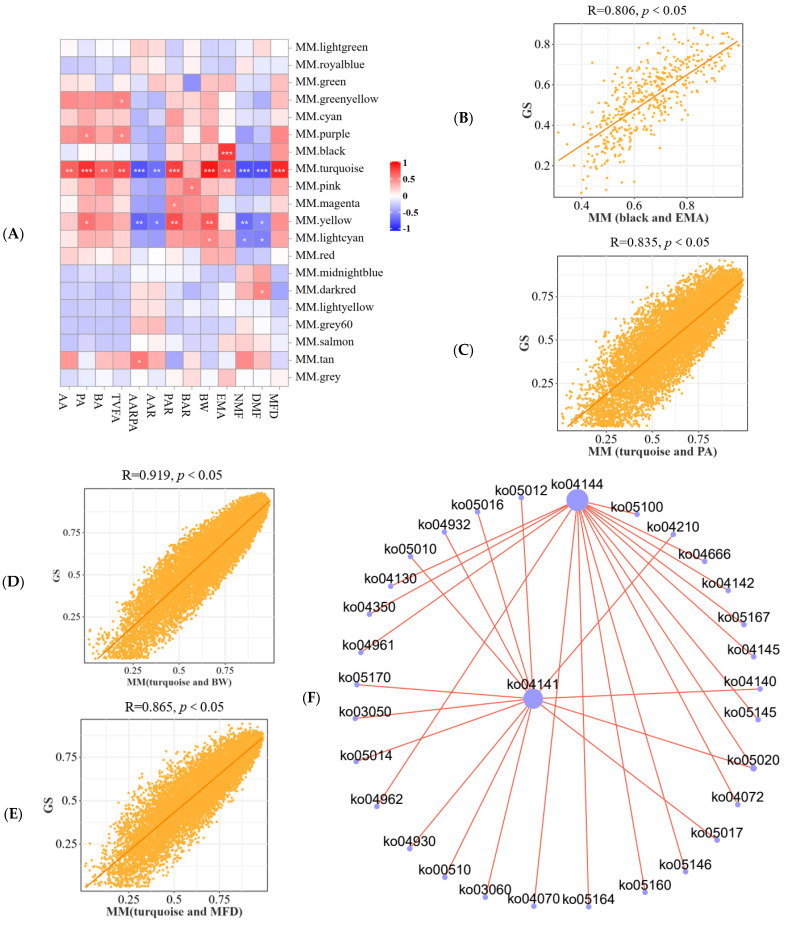

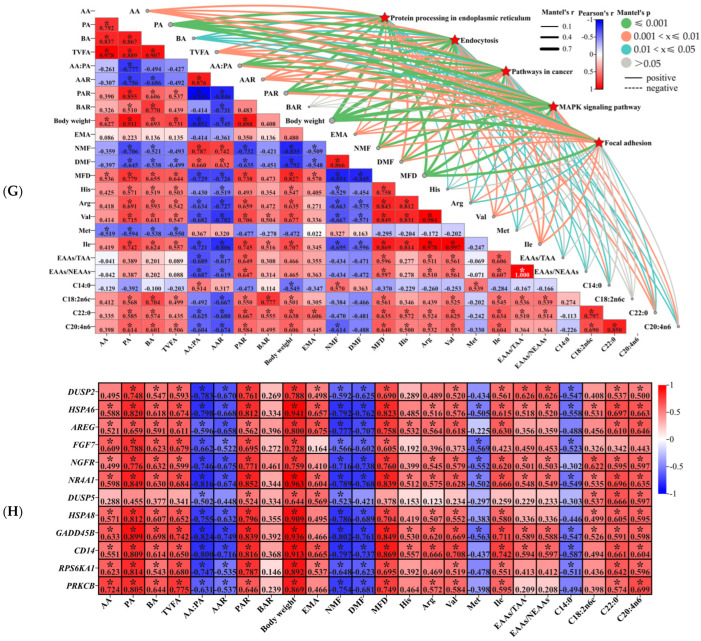
Analysis host phenotype and gene module association of the HADG and LADG lambs. (**A**) Heatmap of longissimus dorsi gene enrichment module and host phenotype correlation in lambs. In the correlation heat map,* for 0.01 ≤ *p* < 0.05, ** for 0.001 ≤ *p* < 0.01, *** for *p* < 0.001. Correlation scatterplots (**B**) (black and EMA), (**C**) (turquoise and PA), (**D**) (turquoise and BW), and (**E**) (turquoise and MFD) of host phenotypes and gene enrichment modules were plotted according to R > 0.8. (**F**) KEGG enrichment network map of genes within the turquoise module. (**G**) Mantel’s r analysis of turquoise module gene KEGG top 5 pathways correlation with phenotype of HADG and LADG lambs. The width of the edges is representative of Mantel’s r statistics associated with the respective distance correlations, while the color of the edges reflects the significance of Mantel’s *p* statistic, where a gray line denotes indicating *p* > 0.05, and other colors line indicating *p* < 0.05. (**H**) MAPK signaling pathway differential genes and host phenotypes correlation in lambs. Note: the values in the correlation heat map indicate the correlation coefficients. * In the correlation heat map, we indicate *p* < 0.05. Abbreviations: AARPA: acetic acid ratio propionic acid; BW: body weight.

As seen in Figure 5, the correlation was closest between the turquoise module and the phenotype and pooled the highest number of genes, followed by the yellow module (Figure 5A and Supplementary Figure S2). Furthermore, the turquoise module was significantly positively correlated with AA, PA, BA, TVFA, PAR, body weight (BW), EMA, and MFD, but significantly negatively correlated with AARPA, AAR, NMF, and DMF (*p* < 0.05) (Figure 5A). In addition, the yellow module was significantly positively correlated with PA, PAR, and BW, but significantly negatively correlated with acetic acid ratio propionic acid, AAR, NMF, and DMF (*p* < 0.05) (Figure 5A). TVFA has significant positive correlation with greenyellow and purple modules (*p* < 0.05) (Figure 5A). The lightcyan module is significantly positively correlated with BW, but significantly negatively correlated with DMF and NMF (*p* < 0.05) (Figure 5A). Furthermore, AARPA and the tan module, BAR and the pink module, PA and the purple module, and PAR and the magenta module were significantly positively correlated (*p* < 0.05) (Figure 5A). It is noteworthy that the correlation coefficients between the black module and EMA (Figure 5B), the turquoise module and PA (Figure 5C), BW (Figure 5D), and MFD (Figure 5E) were all greater than 0.8.

Subsequently, we mapped the transcripts within the turquoise module to the KEGG database and plotted the top 30 enrichment bars based on FDR values, including protein processing in endoplasmic reticulum, endocytosis, pathways in cancer, MAPK signaling pathway, focal adhesion, etc., as illustrated in Supplementary Figure S3. Additionally, protein processing in endoplasmic reticulum and endocytosis are closely related to other pathways (Figure 5F). Furthermore, our correlation analysis of the KEGG top 5 metabolic pathways in the turquoise module with body weight, rumen VFA, and muscle differential phenotypes revealed a significant effect on body weight, rumen VFA (AA, PA, BA, TVFA, AA:PA, AAR, and PAR), EMA, muscle fiber (NMF, DMF, MFD), longissimus dorsi amino acid (His, Arg, Val, Met, Ile, EAAs/TAA, and EAAs/NEAAs), and fatty acid (C14:0, C22:0, and C20:4n6) (Figure 5G), specifically, and body weight (Figure 5D,G), PA (Figure 5C,G), and MFD (Figure 5E,G). In addition, differential genes in the MAPK signaling pathway and host phenotypes were significantly correlated, especially with body weight. (Figure 5H).

### 3.6. Analysis of Metabolism Profiling of Longissimus Dorsi

**Figure 6:**
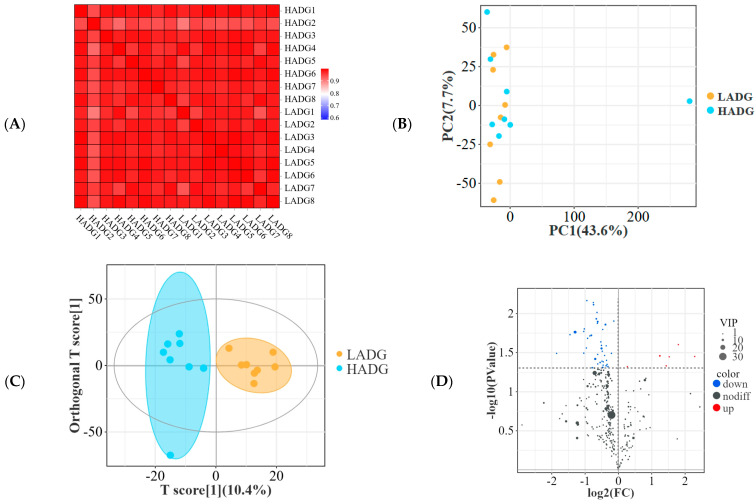

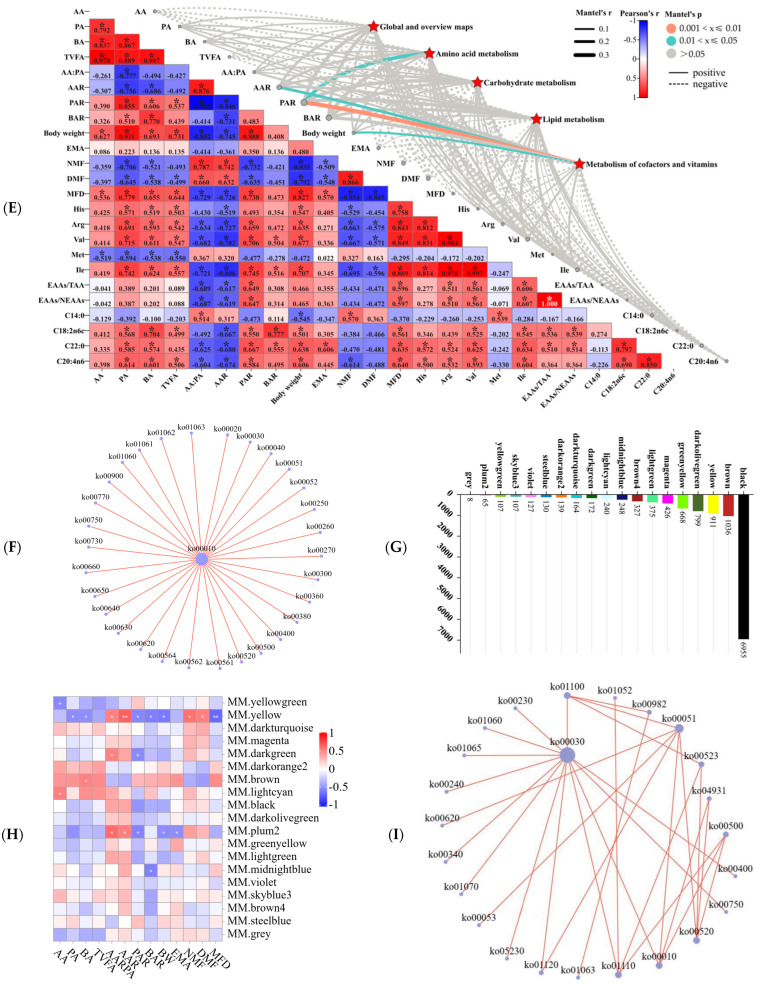

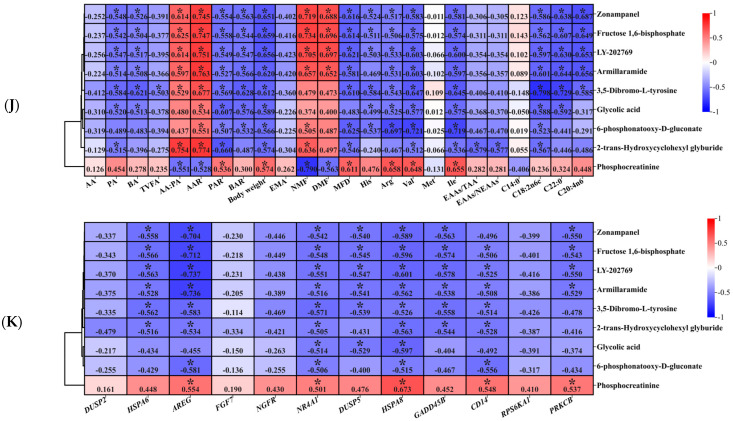
Analysis of metabolism profiling of longissimus dorsi of HADG and LADG lambs. (**A**) Heatmap of correlation between HADG and LADG lambs’ longissimus dorsi samples. Longissimus dorsi metabolism samples PCoA analysis (**B**), OPLS-DA analysis (**C**), and volcano plots (**D**) of HADG and LADG lambs. (**E**) Mantel’s r analyzed the correlation between the longissimus dorsi five KEGG pathways with the highest number of metabolite enrichment and lamb phenotype. The edge width corresponds to the Mantel’s r statistic for the corresponding distance correlations, and the edge color indicates the significance of the Mantel’s *p* statistic, with a gray line indicating *p* > 0.05, and other colors line indicating *p* < 0.05. (**F**) Correlation network diagram between KEGG-enriched metabolic pathways. A histogram (**G**) shows the number of metabolites enriched in back muscle modules, along with a heat map of correlations between these modules and lamb phenotypes (**H**). In the correlation heat map, * for 0.01 ≤ *p* < 0.05, ** for 0.001 ≤ *p* < 0.01. (**I**) Network diagram of correlation between KEGG enrichment pathways in the yellow module. Correlation analysis of longissimus dorsi yellow module differential metabolites and host phenotypes (**J**) and MPAK signaling pathway differential genes (**K**). Note: the values in the correlation heat map indicate the correlation coefficients. * In the correlation heat map, we indicate *p* < 0.05. Abbreviations: HADG: highest average daily gain; LADG: lowest average daily gain.

It can be seen from the heatmap sample (R > 0.910, Figure 6A), PCoA (Figure 6B) and OPLS-DA (Figure 6C) that there were differences in the groups of samples that could be analyzed subsequently. Next, volcano plots according to VIP > 1 and *p* < 0.05 containing 48 (6 up-regulated and 42 down-regulated) differential metabolites (Figure 6D). Next, our analysis of the correlation between the five most enriched metabolic pathways in metabolites found in the longissimus dorsi (Supplementary Figure S4) and different host phenotypes—including body weight, rumen VFAs, and muscle phenotypes—revealed that there was no significant impact on the rumen VFAs (AA, PA, BA, TVFA, and AA:PA), EMA, muscle fiber (NMF, DMF, MFD), longissimus dorsi amino acid (His, Arg, Val, Met, Ile, EAAs/TAA, and EAAs/NEAAs), and fatty acid (C14:0, C18:2n6c, C22:0, and C20:4n6) (Figure 6E). However, significant effects were observed on AAR, PAR, and body weight (Figure 6E).

Furthermore, we plotted KEGG enrichment top 30 histograms (Supplementary Figure S5), and network diagrams (Figure 6F) based on FDR values in which metabolites were mainly enriched in Glycolysis/Gluconeogenesis, TCA cycle, the pentose phosphate pathway, pentose and glucuronate interconversions, and fructose and mannose metabolism. Moreover, Glycolysis/Gluconeogenesis (ko00010) is closely related to other pathways (Figure 6F). Furthermore, we constructed metabolize modules utilizing a power value of 8, with a minimum of 50 metabolize per module and a maximum of 20 modules generated (Figure 6G). Meanwhile, correlation analysis between metabolic modules, lamb body weight, carcass differential phenotypes, and rumen VFAs was performed as shown in Figure 6H. Among them, the relationship between the yellow module and differential phenotypes was the closest (Figure 6H). Subsequently, we plotted yellow module KEGG enrichment histograms (Supplementary Figure S6) and network diagrams (Figure 6I) based on FDR values in which metabolites were mainly enriched in the pentose phosphate pathway, type I polyketide structures, drug metabolism–cytochrome P450, fructose and mannose metabolism, polyketide sugar unit biosynthesis, insulin resistance, starch and sucrose metabolism, etc. (Supplementary Figure S6). In addition, the pentose phosphate pathway (ko00030) is closely related to other pathways (Figure 6I).

Additionally, we found a significant correlation between yellow module differential metabolites and host differential phenotypes (Figure 6J). Among them, the longissimus dorsi up-regulated metabolite phosphocreatinine was significantly positively correlated with PAR, body weight, MFD, Arg, Val, and Ile, but significantly negatively correlated with AA:PA, AAR, NMF, and DMF (Figure 6J). Additionally, longissimus dorsi down–regulated metabolites (zonampanel, fructose 1,6–bisphosphate, LY–202769, armillaramide, 3,5–Dibromo–L–tyrosine, glycolic acid, 6–phosphonatooxy–D–gluconate, and 2–trans–Hydroxycyclohexyl glyburide) were overall significantly positively correlated with AA:PA, AAR, NMF, and DMF, but it is overall significantly negatively correlated with PA, BA, PAR, BAR, body weight, MFD, His, Arg, Val, Ile, and C18:2n6c (Figure 6J). Overall, MPAK signaling pathway differential genes (*HSPA6*, *AREG*, *NR4A1*, *DUSP5*, *HSPA8*, *GADD45B*, *CD14*, and *PRKCB*) in the turquoise module of the longissimus dorsi transcript were negatively correlated with metabolites down-regulated in the metabolic profile yellow module (Figure 6K). In addition, *AREG*, *NR4A1*, *HSPA8*, *CD14*, and *PRKCB* was significantly and positively correlated with phosphocreatinine (Figure 6K).

### 3.7. Liver Metabolism Profiling and Rumen Microbe–Muscle–Liver Interactions Analysis

**Figure 7:**
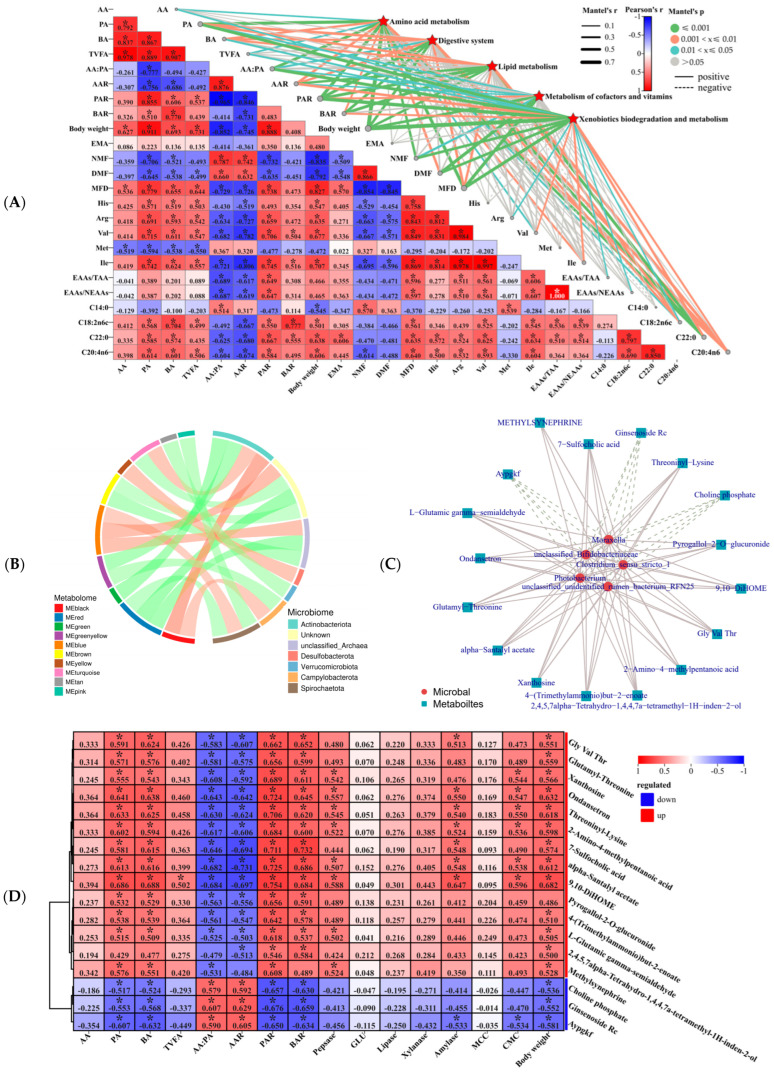

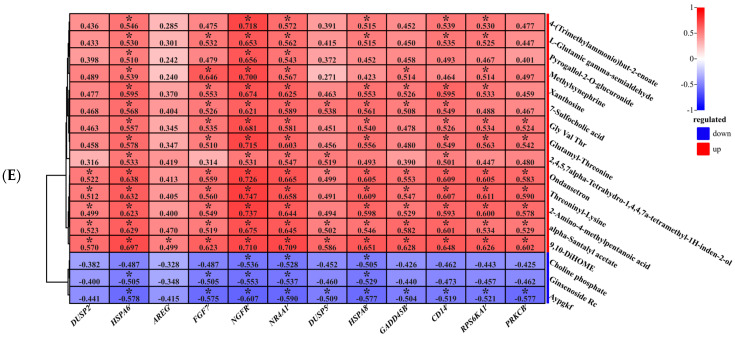
Liver metabolism profiling and rumen microbe–muscle–liver interactions analysis of HADG and LADG lambs. (**A**) Mantel’s r analyzed the correlation between the five KEGG pathways with the highest number of liver metabolite enrichment and lamb phenotype. The edge width corresponds to the Mantel’s r statistic for the corresponding distance correlations, and the edge color indicates the significance of the Mantel’s *p* statistic, with a gray line indicating *p* > 0.05, and other colors line indicating *p* < 0.05. (**B**) Liver metabolic modules and rumen microbial chord diagrams. (**C**) Liver differential metabolism and rumen differential microbial correlation analysis. Solid lines indicate positive correlations and dashed lines indicate negative correlations. (**D**) Heatmap of correlation between liver–microbe-related differential metabolites and lamb phenotype. (**E**) Heatmap of correlation between liver–microbe related differential metabolites and longissimus dorsi developmentally relevant signaling pathway differential genes. Note: the values in the correlation heat map indicate the correlation coefficients. * In the correlation heat map, we indicate *p* < 0.05.

Overall, liver differential metabolites were correlated with rumen VFA molar content (AA, PA, BA, TVFA), VFA molar ratio (AA:PA, AAR, PAR, and BAR), body weight, muscle fiber (NMF, DMF, MFD), longissimus dorsi amino acid (Arg, Val, Ile, EAAs/TAA, and EAAs/NEAAs), and fatty acid (C18:2n6c, C22:0, and C20:4n6) were significantly correlated (Figure 7A). Moreover, AA, PA, BA, TVFA, PAR, BAR, body weight, EMA, MFD, His, Arg, Val, Ile, EAAs/TAA, EAAs/NEAAs, C18:2n6c, C22:0, and C20:4n6 and liver up-regulated metabolite (such as 9,10–DHOME, 2,3–Dinor–8–iso prostaglandin F2alpha, cholic acid, succinic acid, choloyl–CoA, betaine, Choline, Estradiol–17beta 3–glucuronide, etc.) were positively correlated, but negatively correlated with down-regulated metabolites (such as arachidonate, calcitriol, 16alpha–Hydroxyestrone, 7alpha–Hydroxycholest–4–en–3–one, 8,9–EET, 6–Keto–prostaglandin F1alpha, etc.) (Supplementary Figure S7). Moreover, AA:PA, AAR, NMF, DMF, Met, and C14:0 and liver down-regulated metabolites were positively correlated, but negatively correlated with up-regulated metabolites (Supplementary Figure S7). Moreover, rumen microbes and liver metabolite enrichment modules are closely related (Figure 7B). In addition, rumen differential microbial *Clostridium_sensu_stricto_1*, *Moraxella*, *Photobacterium*, *unclassified_Bifidobacteriaceae*, and *unclassified_unidentified_rumen_bacterium_RFN25* were positively correlated with liver up-regulated metabolites (alpha–Santalyl acetate, 2,4,5,7alpha–Tetrahydro–1,4,4,7a–tetramethyl–1H–inden–2–ol, 9,10–DiHOME, 7–Sulfocholic acid, Glutamyl–Threonine, Gly Val Thr, L–Glutamic gamma–semialdehyde, 2–Amino–4–methylpentanoic acid, Threoninyl–Lysine, Ondansetron, 4–(Trimethylammonio)but–2–enoate, Xanthosine, Pyrogallol–2–O–glucuronide, and methylsynephrine) and negatively correlated with down-regulated (Aypgkf, Ginsenoside Rc, and Choline phosphate) metabolites (Figure 7C). In addition, liver and rumen microbes were significantly correlated and up-regulated metabolites in liver showed significant positive correlation with body weight, rumen VFAs (PA, BA, PAR, and BAR), longissimus dorsi turquoise module enriched in MAPK signaling pathway differential genes (*HSPA6*, *FGF7*, *NGFR*, *NR4A1*, *HSPA8*, *CD14*, and *RPS6KA1*), but significant negative correlation with AA:PA and AAR (Figure 7D,E).

## 4. Discussion

Lamb weight gain involves increases in tissue mass and nutrient deposition influenced by genetics, environment, nutrition, and gastrointestinal microbiota [7,15,16]. The body weight of lambs is a crucially economic trait, and studies have demonstrated that animals with an elevated Kleiber ratio are considered effective feed users [34,35]. The results of this experiment indicate that different growth and development levels of Hu lambs, despite being subjected to the same feeding and management conditions, did significantly impact body weight, eye muscle area, and muscle fibers. This result may be attributed to the significant coefficient of variation in body weight among individuals in Hu lamb, as well as the influence of their paternal microbiome on the productive performance of the offspring [46,47]. However, the differences in muscle meat quality, transcriptional metabolism, rumen function, and liver metabolism in lambs with varying Kleiber ratios require further investigation. Furthermore, eye muscle area, back fat thickness, and rib thickness are critical indicators of lamb production performance and carcass quality. Specifically, carcass lean yield decreases with increasing back fat thickness, and rib thickness, while it increases with a larger eye muscle area [48,49,50,51]. We compared carcass and meat physical characteristics and nutritional composition of lambs at different levels of development. This study found that HADG lambs had a significantly larger eye muscle area compared to LADG lambs, while no significant differences were observed in back fat thickness, and rib thickness. Additionally, the eye muscle area showed a significant positive correlation with MFD and a significant negative correlation with DMF and NMF. There were no significant differences in physical traits, such as longissimus dorsi quality, conventional nutrients, TAA, NEAAs, EAAs, SFA, and UFA among lambs at different levels of growth and development. This phenomenon may be attributed to the influence of several factors on animal-derived meat, including genetics, nutrition, age, sex, and physical activity [48,49]. Research indicates that specific amino acids, including tryptophan, threonine, arginine, lysine, and leucine, significantly influence the taste of mutton [52]. Additionally, amino acid metabolism, particularly involving arginine and proline, histidine, and tryptophan metabolism, is crucial for regulating meat quality [49]. The results of this experiment indicate that HADG lambs are able to increase the nutritional value of muscle and have the potential to improve muscle taste by increasing histidine, arginine, valine, isoleucine, EAAs/TAA, andEAAs/NEAAs in the longissimus dorsi [49,53]. This result may be related to differences in the gluconeogenic pathway in lambs at different levels of growth and development, such as significantly increased *PCK1* expression in muscle, rumen propionic acid and propionic acid ratio [54,55,56,57]. Studies have demonstrated that PCK1, a key enzyme in gluconeogenesis, improves amino acid utilization efficiency, and its rise coincided with increased essential amino acid content [54,55]. Moreover, propionic acid enhances the expression of key gluconeogenesis genes through the gut–brain axis [57], and increasing propionic acid production nutritional interventions that increase propionic acid production may improve hepatic gluconeogenesis and ruminant performance [56]. Fatty acids in muscle are essential for taste development and an important indicator of meat quality [58], such as when high SFA (myristic acid and palmitic acid) in meat affect cholesterol metabolism, increase the risk of cardiovascular disease [59,60]. There was no significant effect of different developmental lambs on the content of SFA and UFA in the longissimus dorsi, but HADG lambs had significantly higher linoleic acid, behenic acid, and arachidonic acid in muscle, whereas myristic acid was significantly reduced. In conclusion, HADG lambs are more beneficial to the health of the organism, including a reduction in myristic acid levels [60], an increase in linoleic acid—a novel functional polyunsaturated fatty acid that is essential and cannot be synthesized by humans or animals [61]—as well as an improvement in arachidonic acid, which has been shown to improve both immunity and reproductive performance in these animals [62].

The microbial fermentation occurring in the rumen of ruminants produces VFAs, which are crucial for the physiological functions of the host. These VFAs account for approximately 70% of the energy requirements of ruminants and are integral to the regulation of metabolic processes in the liver and skeletal muscles [7,19,20,21]. It was shown that HADG lambs significantly increased rumen digestive enzymes and improved rumen efficiency. Furthermore, body weight was significantly and positively correlated with AA, PA, BA, TVFAs, PAR, pepsase, xylanase, amylase, and CMC, and especially strongly correlated with PA, PAR, pepsase, and CMC, but significantly and negatively correlated with AA:PA and AAR. The findings align with those of Wang et al., indicating that the average daily gain in 180 days of age goats is positively correlated with the levels of PA, BA, and PAR in rumen fluid, while showing a negative correlation with AAR and AA:PA [63]. Furthermore, elevated levels of propionic and butyric acid were correlated with enhanced feed efficiency [64]. Thus, in livestock production, enhancing the content and ratio of propionic and butyric acids while reducing the acetic acid to propionic acid ratio can be achieved through nutritional strategies, such as incorporating grain feeds [65] and monensin [66,67] to promote the growth and development of fattening lambs. Additionally, the ability of lambs to digest plant-based feeds is influenced by the microbial community present in their gastrointestinal tract [2], which is linked to feed efficiency [8,9] and body weight [7]. Consequently, we hypothesized that variations in rumen VFAs and digestive enzymes in lambs, which differ in growth and development level, may be linked to the composition of their microbiota [7,22,23,68].

Subsequently, analysis of the rumen microbial community in lambs revealed that the rumen was dominated by Firmicutes and Bacteroidetes. These findings align with previous research indicating that the primary rumen microbial populations in Hu lambs are also Firmicutes and Bacteroidetes [39,69]. The present experiment revealed ruminal propionic acid and *Prevotella* abundance increase in HADG lambs, which may be related to the study that demonstrated that elevated Kleiber ratio [34,35] and propionic acid content are associated with better feed efficiency [64]. In addition, *Prevotella* is essential for carbohydrate and hydrogen metabolism, with genes that enable the processing of complex carbohydrates and polysaccharides for propionate synthesis, the main substrate for hepatic gluconeogenesis in ruminants [70,71]. Moreover, *Prevotella* enhances nutrient biosynthesis in ruminants and reduces the environmental impact of rumen metabolism, such as *Prevotella* redirects hydrogen flow during glycolysis from methanogenesis to propionate production, which decreases the hydrogen required for methane by methanogens [71]. Furthermore, the construction of a genus level top 80 rumen microbial correlation network map revealed that firmicutes dominated in both HADG and LADF lambs, supporting previous research on the pronounced correlation among rumen firmicutes microbiota [15,69], and that firmicutes contain genes encoding enzymes related to energy metabolism [72]. Competitive interactions of microbiota predominate and are more ecologically important than synergistic interactions, such as reciprocal or attachment symbioses [3]. Moreover, Mantel’s analysis found no significant correlations between rumen microbes and lamb rumen VFAs, digestive enzymes, and carcass traits. This result may arise from Mantel’s r focusing on linear correlations, neglecting nonlinear relationships that could impact correlations [7]. Furthermore, complex microbiota interactions may be more crucial to ecosystem function than abundance in complex rumen ecosystems [63,73]. Consequently, when focusing the physiological functions of dominant bacterial populations, the potential contributions of low-abundance bacteria exhibiting greater taxonomic diversity to host functions warrant increased attention [74,75].

The longissimus dorsi of different growth and development levels were collected for analyses transcriptome and metabolome to explore molecular factors affecting lamb development. There is an increasing body of evidence suggesting that the gut microbiota may play a significant role in influencing muscle metabolic processes [4,5,6]. Thus, changes in molar concentrations and ratios of VFAs induced by the microbiological composition of the lamb rumen may modulate muscle development and meat quality [6,10,11,76]. Studies have demonstrated that a number of genes can be candidates for muscle growth and development, such as *PCK1* [77], *SPP1* [78], *FGF7* [79,80], *NR4A1* [81,82], *DUSP5* [83], and *GADD45B* [84]. This study revealed significant differences in dorsal muscle transcripts among lambs at different growth levels, and the significant differences in genes and body weight are strongly correlated, especially *SPP1*. The *SPP1* is identified as potentially significant in regulating muscle growth and development, making it a candidate gene for enhancing growth traits in sheep breeding [78,85]. Our correlation analysis indicated that the longissimus dorsi genes were mainly enriched in the turquoise module and significant correlations with lamb phenotypes, particularly demonstrating strong associations with PA, BW, and MFD. Furthermore, the MAPK signaling pathway plays a crucial role in muscle growth and development [86], such as *FGF7* [79,80], *NR4A1* [81,82], *DUSP5* [83], and *GADD45B* [84]. The present experiment found a significant correlation between body weight and *FGF7* (R = 0.728), *NR4A1* (R= 0.963), *DUSP5* (R = 0.644)*,* and *GADD45B* (R = 0.936). Moreover, the KEGG top 5 metabolic pathways in the turquoise module was significantly associated with body weight, rumen VFA, and muscle differential phenotype, specifically and body weight, PA, and MFD. Additionally, skeletal muscle is a significant metabolic organ of the organism, playing a crucial role in regulating the metabolic functions of other tissues, including the pancreas and liver [27].

Subsequently, our metabolomic analysis of muscle in lambs at different growth and development levels revealed that Glycolysis/Gluconeogenesis (ko00010) is closely related to other pathways. However, the top 5 pathways with the highest muscle metabolite enrichment were not significantly correlated with host difference phenotypes. This result may be related to the Mantel’s r focusing on linear correlations, neglecting nonlinear relationships that could impact correlations [7], and the interplay between muscle metabolism and the metabolism of target tissues such as the pancreas and liver [27,31,87]. Meanwhile, our correlation analysis indicated that the longissimus dorsi metabolic were mainly enriched in the black module, but with differential phenotypes the closest was the yellow module. This study showed that phosphocreatinine maintains high ATP levels in skeletal muscle and is essential for energy homeostasis in both skeletal and cardiac muscles [88]. Furthermore, the longissimus dorsi upregulated metabolite phosphocreatinine was significantly positively correlated with PAR, body weight, MFD, Arg, Val, and Ile, but significantly negatively correlated with AA:PA, AAR, NMF, and DMF. Results from the muscle metabolome suggest that skeletal muscle plays an important function in animal energy homeostasis [27,31,87,88]. Concurrently, skeletal muscle possesses the ability to influence the metabolic functions of hepatic tissue, whereas hepatic metabolic factors play a crucial role in mediating interactions between the liver and other organs in the body [27,30,32]. This study showed that liver metabolite enrichment modules and rumen microbes are closely related. Furthermore, amino acid metabolism is crucial for metabolic precursor formation, meat quality regulation in sheep, and the promotion of epigenetic modifications, especially glycine, serine, and threonine metabolism, tyrosine metabolism, arginine and proline metabolism, cysteine and methionine metabolism, and phenylalanine metabolism [49,89]. Meanwhile, liver enrichment of differential metabolites in the top 5 (amino acid metabolism, digestive system, lipid metabolism, metabolism of cofactors and vitamins, and xenobiotics biodegradation and metabolism) pathway overall and rumen VFA (AA, PA, BA, TVFA, AA:PA, AAR, PAR, and BAR), body weight, muscle fiber (NMF, DMF, MFD), longissimus dorsi amino acid (Arg, Val, Ile, EAAs/TAA, and EAAs/NEAAs), and fatty acid (C18:2n6c, C22:0, and C20:4n6) were significantly correlated. Thus, VFAs produced by rumen fermentation may influence lamb growth by modulating liver metabolic homeostasis and may influence lamb growth by regulating lipid and amino acid metabolism in animals [21,22,56,90]. Furthermore, we found an overall positive correlation between liver upregulation of differential metabolites (such as spermine, cholic acid, succinic acid, betaine, etc.) and a significantly higher phenotype in HADG lambs (such as PA, BA, TVFA, PAR, body weight, and MFD). These findings suggest that small molecule compounds like VFAs, bile acids, succinic acid, and betaine can affect key signaling molecules related to animal growth and development in microbe–host interactions, and that they have the potential to improve animal growth performance [7,21,22,23,24,25,26]. In summary, skeletal muscle plays a crucial role in regulating the metabolic functions of liver tissue, and liver metabolic factors significantly contribute to the interactions between the liver and other organs and tissues within the body, such as muscle and gastrointestinal microbiota, while also influencing lamb growth and development [22,27,30,32].

## 5. Conclusions

This study on HADG lambs revealed significant improvements in muscle fiber diameter, eye muscle area, and body weight, improved amino acid (histidine, arginine, valine, isoleucine, essential amino acid/total amino acid, and essential amino acid/nonessential amino acid) and fatty acid (Cis–Linoleate acid, behenic acid, and arachidonic acid) composition. It also noted an increased activity of rumen enzymes (pepsase, lipase, xylanase, amylase, and carboxymethyl cellulose) and fermentation parameters (acetic acid, propionic acid, isobutyric acid, butyric acid, valeric acid, TVFA, and propionic acid ratio), with a notable rise in the rumen microbe *Prevotella*. Correlations were found between specific rumen markers (*Schwartzia* and *Streptococcus*) and meat quality traits, while liver and muscle metabolic profile analysis indicated that their host–differential phenotypes were closely related. Furthermore, muscle transcriptome analysis indicated that the turquoise module significantly correlated with the host phenotype, especially regarding body weight. In conclusion, rumen microbe–muscle–liver interactions in lambs enhance rumen fermentation, regulating muscle transcriptional, altering liver and muscle metabolism profiles, thereby facilitating adaptation to lamb growth and development, identifying potential molecular targets for improving lamb production. This finding provides a theoretical basis to further exploit the production potential of Hu lambs.

## Figures and Tables

**Table 1:** Analysis of carcass and meat physical traits in lambs.

Items		LADG	HADG	*p*-Value
Body weight, Kg		25.80 ± 0.46 ^b^	40.91 ± 0.78 ^a^	<0.001
Back fat thickness, mm		2.46 ± 0.26	2.77 ± 0.26	0.406
Rib thickness (GR), mm		4.70 ± 0.44	6.15 ± 0.72	0.108
Eye muscle area (EMA), mm^2^		950.61 ± 78.82 ^b^	1456 ± 175.73 ^a^	0.020
Meat color (45 min)	a*	75.07 ± 0.57	73.17 ± 0.95	0.113
	b*	24.91 ± 0.60	23.71 ± 0.41	0.118
	L*	44.44 ± 1.34	42.19 ± 2.09	0.379
pH (45 min)		6.58 ± 0.07	6.51 ± 0.07	0.479
Shear force, N		64.15 ± 4.09	72.54 ± 2.87	0.115
Water loss rate, %		5.37 ± 0.58	6.90 ± 1.43	0.347
Cooking loss, %		63.53 ± 1.08	59.11 ± 2.36	0.111
Muscle fiber diameter (MFD), mm		0.03 ± 0.00 ^b^	0.04 ± 0.00 ^a^	<0.001
Number of muscle fibers (NMF), n		213.25 ± 9.90 ^a^	154.88 ± 6.43 ^b^	<0.001
Density of muscle fibers (DMF), n/mm^2^		1250.6 ± 75.72 ^a^	868.49 ± 34.34 ^b^	<0.001

Abbreviations: HADG: highest average daily gain; LADG: lowest average daily gain; a*: redness; b*: yellowness; L*: lightness. Note: The presence of different lowercase superscript letters in the same row of the table indicates a significant difference between groups, *p* < 0.05.

**Table 2:** Analysis of the base nutritional components of the longissimus dorsi in lambs.

Items		LADG	HADG	*p*-Value
Base nutritional components	Crude protein, %	20.03 ± 0.42	20.57 ± 0.36	0.349
	Moisture, %	73.06 ± 0.32	72.60 ± 0.47	0.432
	Crude fat, %	4.81 ± 0.43	3.21 ± 0.65	0.058
Amino acids	Aspartate (Asp), g/100 g	1.53 ± 0.09	1.80 ± 0.15	0.153
	Glutamic acid (Glu), g/100 g	2.54 ± 0.13	2.98 ± 0.25	0.141
	Serine (Ser), g/100 g	0.56 ± 0.03	0.60 ± 0.05	0.407
	Glycine (Gly), g/100 g	0.42 ± 0.03	0.46 ± 0.06	0.538
	Histidine (His), g/100 g	0.37 ± 0.05 ^b^	0.53 ± 0.06 ^a^	0.043
	Arginine (Arg), g/100 g	0.95 ± 0.06 ^b^	1.31 ± 0.13 ^a^	0.021
	Alanine (Ala), g/100 g	0.68 ± 0.03	0.76 ± 0.07	0.292
	Proline (Pro), g/100 g	0.26 ± 0.04	0.32 ± 0.03	0.279
	Tyrosine (Tyr), g/100 g	0.45 ± 0.02	0.54 ± 0.05	0.081
	Valine (Val), g/100 g	0.41 ± 0.02 ^b^	0.60 ± 0.06 ^a^	0.016
	Methionine (Met), g/100 g	0.14 ± 0.03 ^a^	0.07 ± 0.02 ^b^	0.047
	Isoleucine (Ile), g/100 g	0.38 ± 0.02 ^b^	0.58 ± 0.06 ^a^	0.010
	Leucine (Leu), g/100 g	0.87 ± 0.05	1.06 ± 0.09	0.089
	Phenylalanine (Phe), g/100 g	0.33 ± 0.02	0.42 ± 0.04	0.070
	Lysine (Lys), g/100 g	1.31 ± 0.07	1.66 ± 0.15	0.063
	Threonine (Thr), g/100 g	0.34 ± 0.02	0.41 ± 0.04	0.106
	TAA, g/100 g	11.55 ± 0.59	14.11 ± 1.26	0.086
	NEAAs, g/100 g	7.75 ± 0.38	9.31 ± 0.83	0.111
	EAAs, g/100 g	3.79 ± 0.21	4.81 ± 0.44	0.055
	EAAs/TAA,%	32.79 ± 0.41 ^b^	34.01 ± 0.32 ^a^	0.034
	EAAs/NEAAs,%	48.82 ± 0.92 ^b^	51.57 ± 0.73 ^a^	0.034
Fatty acid	Caproic acid (C6:0), mg/100 g	5.33 ± 0.34	7.08 ± 1.20	0.199
	Caprylic acid (C8:0), mg/100 g	5.01 ± 0.46	5.51 ± 0.42	0.435
	Undecanoic acid (C11:0), mg/100 g	15.95 ± 0.51	14.84 ± 0.40	0.109
	Myristic acid (C14:0), mg/100 g	57.38 ± 11.66 ^a^	28.44 ± 4.07 ^b^	0.034
	Pentadecanoic acid (C15:0), mg/100 g	7.97 ± 1.46	11.98 ± 3.82	0.344
	Palmitic acid (C16:0), mg/100 g	441.10 ± 71.39	286.80 ± 37.27	0.076
	Palmitoleic acid (C16:1), mg/100 g, mg/100 g	32.52 ± 6.22	29.13 ± 4.07	0.655
	Margaric acid (C17:0), mg/100 g	20.58 ± 2.97	14.73 ± 2.47	0.152
	Margaroleic acid (C17:1), mg/100 g	14.81 ± 1.99	16.75 ± 2.54	0.558
	Stearic acid (C18:0), mg/100 g	435.71 ± 65.31	368.03 ± 55.30	0.442
	Oleic acid (C18:1n9c), mg/100 g	1017.05 ± 166.84	682.8 ± 97.38	0.106
	linoleic acid (C18:2n6c), mg/100 g	64.29 ± 6.38 ^b^	86.04 ± 6.01 ^a^	0.026
	γ–linolenic acid (C18:3n6), mg/100 g	22.57 ± 3.22	30.73 ± 4.55	0.165
	ɑ–Linolenic acid (C18:3n3), mg/100 g	8.78 ± 0.82	10.40 ± 0.67	0.150
	Heneicosanoic acid (C21:0), mg/100 g	6.52 ± 1.11	5.37 ± 0.62	0.379
	Behenic acid (C22:0), mg/100 g	11.16 ± 0.91 ^b^	16.20 ± 1.06 ^a^	0.003
	Arachidonic acid (C20:4n6), mg/100 g	45.19 ± 2.95 ^b^	64.45 ± 5.41 ^a^	0.007
	Tricosanoic acid (C23:0), mg/100 g	15.98 ± 0.70	16.91 ± 1.08	0.481
	SFA, mg/100 g	1022.70 ± 151.96	775.90 ± 95.19	0.190
	UFA, mg/100 g	1205.22 ± 173.18	920.31 ± 98.91	0.175

Abbreviations: HADG: highest average daily gain; LADG: lowest average daily gain; TAA: total amino acid; NEAAs: nonessential amino acids; EAAs: essential amino acids; SFA: saturated fatty acid; UFA: unsaturated fatty acid. Note: The presence of different lowercase superscript letters in the same row of the table indicates a significant difference between groups, *p* < 0.05.

**Table 3:** Analysis of rumen VFAs in LADG and HADG lambs.

Items		LADG	HADG	*p*-Value
VFA molar concentration, mmol/L	AA	33.44 ± 3.90 ^b^	48.17 ± 3.16 ^a^	0.011
	PA	5.48 ± 0.33 ^b^	13.40 ± 1.13 ^a^	<0.001
	IBA	0.76 ± 0.04 ^b^	1.05 ± 0.08 ^a^	0.011
	BA	5.72 ± 0.84 ^b^	10.33 ± 1.10 ^a^	0.005
	IVA	1.10 ± 0.06	1.35 ± 0.14	0.123
	VA	0.46 ± 0.05 ^b^	0.92 ± 0.17 ^a^	0.032
	TVFA	46.92 ± 4.98 ^b^	75.22 ± 5.22 ^a^	0.002
VFA molar proportion, %	AA:PA	6.03 ± 0.37 ^a^	3.68 ± 0.21 ^b^	<0.001
	AAR	70.92 ± 0.75 ^a^	64.29 ± 1.61 ^b^	0.002
	PAR	12.00 ± 0.56 ^b^	17.71 ± 0.62 ^a^	<0.001
	IBAR	1.72 ± 0.17	1.39 ± 0.07	0.088
	BAR	11.94 ± 0.54	13.63 ± 1.08	0.184
	IVAR	2.50 ± 0.25 ^a^	1.79 ± 0.11 ^b^	0.022
	VAR	1.04 ± 0.14	1.19 ± 0.16	0.468

Abbreviations: HADG: highest average daily gain; LADG: lowest average daily gain; AA: acetic acid; PA: propionic acid; BA: butyric acid; IBA: isobutyric acid; IVA: isovaleric acid; VA: valeric acid; TVFA: total volatile fatty acids; AAR: acetic acid ratio; PAR: propionic acid ratio; BAR: butyric acid ratio; AA:PA: acetic acid ratio propionic acid; IBAR: isobutyric acid ratio; IVAR: isovaleric acid ratio. Note: The presence of different lowercase superscript letters in the same row of the table indicates a significant difference between groups, *p* < 0.05.

**Table 4:** Analysis of rumen digestive enzyme and histomorphology in LADG and HADG lambs.

Items		LADG	HADG	*p*-Value
Digestive enzyme	Pepsase, ug/L	15.42 ± 0.46 ^b^	20.90 ± 0.62 ^a^	<0.001
	GLU, ng/L	921.04 ± 51.12	897.04 ± 59.29	0.764
	Lipase, ng/mL	237.98 ± 13.77 ^b^	298.72 ± 20.85 ^a^	0.029
	Xylanase, pg/mL	130.77 ± 12.45 ^b^	184.29 ± 8.01 ^a^	0.003
	Amylase, umol/L	141.35 ± 3.87 ^b^	172.99 ± 5.09 ^a^	<0.001
	MCC, pg/mL	114.58 ± 4.77	119.95 ± 5.72	0.483
	CMC, pg/mL	249.06 ± 5.10 ^b^	323.02 ± 7.82 ^a^	<0.001
Histomorphology	Papilla height, mm	2.15 ± 0.24	1.71 ± 0.05	0.112
	Papilla width, mm	0.47 ± 0.05	0.41 ± 0.02	0.361
	Muscle layer, mm	1.46 ± 0.14	1.65 ± 0.17	0.402
	Stratum corneum, mm	0.04 ± 0.00	0.05 ± 0.00	0.399
	Basal layer thickness, mm	0.02 ± 0.00	0.02 ± 0.00	0.452
	Stratum granular, mm	0.01 ± 0.00	0.01 ± 0.00	0.181
	Stratum spinosum, mm	0.08 ± 0.01	0.06 ± 0.00	0.062

Abbreviations: HADG: highest average daily gain; LADG: lowest average daily gain; GLU: beta glucosidase; MCC: microcrystalline cellulase; CMC: carboxymethyl cellulase. Note: The presence of different lowercase superscript letters in the same row of the table indicates a significant difference between groups, *p* < 0.05.

**Table 5:** Analysis of rumen microbial alpha diversity in lambs.

Items	LADG	HADG	*p*-Value
ACE	577.37	582.64	0.88
Chao1	576.16	581.69	0.88
Simpson	0.99	0.98	0.13
Shannon	8.00	7.61	0.19

Abbreviations: HADG: highest average daily gain; LADG: lowest average daily gain; ACE: abundance–based coverage estimator; Chao1: Chao1 richness; Simpson: Simpson’s index; Shannon: Shannon’s diversity index.

## Data Availability

The original contributions presented in this study are included in the article/Supplementary Material. Further inquiries can be directed to the corresponding author.

## Supplementary Materials

The following supporting information can be downloaded at: https://www.mdpi.com/article/10.3390/microorganisms13040943/s1, Supplementary Table S1: Correlation analysis of differential genes and body weight in longissimus dorsi muscle; Supplementary Figure S1: LEfSe analysis rumen biomarkers in HADG and LADG lambs; Supplementary Figure S2: Histogram of the number of lamb longissimus dorsi genes enriched in different modules; Supplementary Figure S3: Bar graph of KEGG top 30 enrichment of turquoise module transcripts; Supplementary Figure S4: longissimus dorsi metabolite KEGG enrichment classification number histograms; Supplementary Figure S5: longissimus dorsi metabolite KEGG–enriched pathway FDR value top 30 histogram; Supplementary Figure S6: longissimus dorsi Yellow modules KEGG–enriched FDR values top 30 histograms; Supplementary Figure S7: Heat maps were used to analyze the correlation between phenotype and major enrichment differences in KEGG regulation in liver.

## Abbreviations

The following abbreviations are used in this manuscript:

AA (AAR)	acetic acid (acetic acid ratio)
BA (BAR)	butyric acid (butyric acid ratio)
CMC	carboxymethyl cellulose
DMF	Density of muscle fibers
EAAs	Essential amino acids
EMA	Eye muscle area
GLU	beta glucosidase
IBA (IBAR)	isobutyric acid (isobutyric acid ratio)
IVA (IVAR)	isovaleric acid (isovaleric acid ratio)
MCC	microcrystalline cellulose
MFD	Muscle fiber diameter
NEAAs	Nonessential amino acids
NMF	Number of muscle fibers
PA (PAR)	propionic acid (acetic acid ratio)
SFA	Saturated fatty acid
TAA	Total amino acid
TVFAs	total volatile fatty acids
UFA	Unsaturated fatty acid
VA (VAR)	valeric acid (valeric acid ratio)
VFAs	volatile fatty acids

## References

[1] Matthews C., Crispie F., Lewis E., Reid M., O’Toole P.W., Cotter P.D. (2019). The rumen microbiome: A crucial consideration when optimising milk and meat production and nitrogen utilisation efficiency. Gut Microbes.

[2] Mizrahi I., Wallace R.J., Moraïs S. (2021). The rumen microbiome: Balancing food security and environmental impacts. Nat. Rev. Microbiol..

[3] Kost C., Patil K.R., Friedman J., Garcia S.L., Ralser M. (2023). Metabolic exchanges are ubiquitous in natural microbial communities. Nat. Microbiol..

[4] Mancin L., Wu G.D., Paoli A. (2023). Gut microbiota–bile acid–skeletal muscle axis. Trends Microbiol..

[5] Frampton J., Murphy K.G., Frost G., Chambers E.S. (2020). Short–chain fatty acids as potential regulators of skeletal muscle metabolism and function. Nat. Metab..

[6] Xu L., Mao T., Xia M., Wu W., Chen J., Jiang C., Zeng T., Tian Y., Lu L., Cai Z. (2024). New evidence for gut–muscle axis: Lactic acid bacteria–induced gut microbiota regulates duck meat flavor. Food Chem..

[7] Wang W., Zhang Y., Zhang X., Li C., Yuan L., Zhang D., Zhao Y., Li X., Cheng J., Lin C. (2023). Heritability and recursive influence of host genetics on the rumen microbiota drive body weight variance in male Hu sheep lambs. Microbiome.

[8] Zhang Y.K., Zhang X.X., Li F.D., Li C., Li G.Z., Zhang D.Y., Song Q.Z., Li X.L., Zhao Y., Wang W.M. (2021). Characterization of the rumen microbiota and its relationship with residual feed intake in sheep. Animal.

[9] Huang Y., Lv H., Song Y., Sun C., Zhang Z., Chen S. (2021). Community composition of cecal microbiota in commercial yellow broilers with high and low feed efficiencies. Poult. Sci..

[10] Dou L., Liu C., Chen X., Yang Z., Hu G., Zhang M., Sun L., Su L., Zhao L., Jin Y. (2023). Supplemental Clostridium butyricum modulates skeletal muscle development and meat quality by shaping the gut microbiota of lambs. Meat Sci..

[11] Wen C., Wang Q., Gu S., Jin J., Yang N. (2024). Emerging perspectives in the gut–muscle axis: The gut microbiota and its metabolites as important modulators of meat quality. Microb. Biotechnol..

[12] Furman O., Shenhav L., Sasson G., Kokou F., Honig H., Jacoby S., Hertz T., Cordero O.X., Halperin E., Mizrahi I. (2020). Stochasticity constrained by deterministic effects of diet and age drive rumen microbiome assembly dynamics. Nat. Commun..

[13] Liu K., Zhang Y., Yu Z., Xu Q., Zheng N., Zhao S., Huang G., Wang J. (2021). Ruminal microbiota–host interaction and its effect on nutrient metabolism. Anim. Nutr..

[14] Yao L., Wang B., Wang Y., Bai J., Gao Y., Ru X., Bi C., Li J., Shan A. (2024). Effects of sex on fat deposition through gut microbiota and short–chain fatty acids in weaned pigs. Anim. Nutr..

[15] Wang H., Zhan J., Jiang H., Jia H., Pan Y., Zhong X., Huo J., Zhao S. (2024). Metagenomics–metabolomics exploration of three–way–crossbreeding effects on rumen to provide basis for crossbreeding improvement of sheep microbiome and metabolome of sheep. Animals.

[16] Kemper K.E., Visscher P.M., Goddard M.E. (2012). Genetic architecture of body size in mammals. Genome Biol..

[17] Li X., Yang J., Shen M., Xie X.L., Liu G.J., Xu Y.X., Lv F.H., Yang H., Yang Y.L., Liu C.B. (2020). Whole–genome resequencing of wild and domestic sheep identifies genes associated with morphological and agronomic traits. Nat. Commun..

[18] McHugh N., Pabiou T., McDermott K., Wall E., Berry D.P. (2018). A novel measure of ewe efficiency for breeding and benchmarking purposes. J. Anim. Sci..

[19] Malmuthuge N., Liang G., Guan L.L. (2019). Regulation of rumen development in neonatal ruminants through microbial metagenomes and host transcriptomes. Genome Biol..

[20] Wahlström A., Sayin S.I., Marschall H.U., Bäckhed F. (2016). Intestinal crosstalk between bile acids and microbiota and its impact on host metabolism. Cell Metab..

[21] Tong A., Li Z., Liu X., Ge X., Zhao R., Liu B., Zhao L., Zhao C. (2024). Laminaria japonica polysaccharide alleviates type 2 diabetes by regulating the microbiota–gut–liver axis: A multi–omics mechanistic analysis. Int. J. Biol. Macromol..

[22] Wang H., Zhan J., Zhao S., Jiang H., Jia H., Pan Y., Huo J. (2024). Interaction between liver metabolism and gut short–chain fatty acids via liver–gut axis affects body weight in lambs. Int. J. Mol. Sci..

[23] van der Hee B., Wells J.M. (2021). Microbial regulation of host physiology by short–chain fatty acids. Trends Microbiol..

[24] Yin Y., Sichler A., Ecker J., Laschinger M., Liebisch G., Höring M., Basic M., Bleich A., Zhang X.J., Kübelsbeck L. (2023). Gut microbiota promote liver regeneration through hepatic membrane phospholipid biosynthesis. J. Hepatol..

[25] Ma S., Wang Y., Chen L., Wang W., Zhuang X., Liu Y., Zhao R. (2024). Parental betaine supplementation promotes gosling growth with epigenetic modulation of IGF gene family in the liver. J. Anim. Sci..

[26] Duan Y., Wang Y., Zhang J., Sun Y., Wang J. (2018). Dietary effects of succinic acid on the growth, digestive enzymes, immune response and resistance to ammonia stress of *Litopenaeus vannamei*. Fish Shellfish Immunol..

[27] Chen Z.T., Weng Z.X., Lin J.D., Meng Z.X. (2024). Myokines: Metabolic regulation in obesity and type 2 diabetes. Life Metab..

[28] Azzu V., Vacca M., Virtue S., Allison M., Vidal-Puig A. (2020). Adipose tissue–liver cross talk in the control of whole–body metabolism: Implications in nonalcoholic fatty liver disease. Gastroenterology.

[29] Yoo E.S., Yu J., Sohn J.W. (2021). Neuroendocrine control of appetite and metabolism. Exp. Mol. Med..

[30] López-Bermudo L., Luque-Sierra A., Maya-Miles D., Gallego-Durán R., Ampuero J., Romero-Gómez M., Berná G., Martín F. (2022). Contribution of liver and pancreatic islet crosstalk to β–cell function/dysfunction in the presence of fatty liver. Front. Endocrinol..

[31] Pedersen B.K., Febbraio M.A. (2012). Muscles, exercise and obesity: Skeletal muscle as a secretory organ. Nat. Rev. Endocrinol..

[32] Jensen-Cody S.O., Potthoff M.J. (2021). Hepatokines and metabolism: Deciphering communication from the liver. Mol. Metab..

[33] Ringseis R., Gessner D.K., Eder K. (2020). The gut–liver axis in the control of energy metabolism and food intake in animals. Annu. Rev. Anim. Biosci..

[34] Ghafouri-Kesbi F., Abbasi M.A., Afraz F., Babaei M., Baneh H., Abdollahi Arpanahi R. (2011). Genetic analysis of growth rate and Kleiber ratio in Zandi sheep. Trop. Anim. Health Prod..

[35] Mehrban H., Naserkheil M., Lee D.H., Ibáñez-Escriche N. (2021). Genetic parameters and correlations of related feed efficiency, growth, and carcass traits in Hanwoo beef cattle. Anim. Biosci..

[36] (2019). Operating Procedures of Livestock and Poultry Slaughtering Sheep and Goat.

[37] Zhan J., Gu Z., Wang H., Liu Y., Wang L., Huang L., Huo J., Wu Y. (2023). Effects of rutin supplementation on growth performance, slaughter performance, serum parameters, and meat quality of Nubian goats. Anim. Sci. J..

[38] Wang H.B., Zhan J.S., Huo J.H., Zhong X.J., Liu Y.H., Zhao S.G. (2022). Effects of dietary rutin on serum immune and antioxidant indices and muscle composition of Hu sheep. Chin. J. Anim. Nutr..

[39] Wang H., Zhan J., Jia H., Jiang H., Pan Y., Zhong X., Zhao S., Huo J. (2024). Relationship between rumen microbial differences and phenotype traits among Hu sheep and crossbred offspring sheep. Animals.

[40] Callahan B.J., McMurdie P.J., Rosen M.J., Han A.W., Johnson A.J., Holmes S.P. (2016). DADA2: High–resolution sample inference from Illumina amplicon data. Nat. Methods.

[41] Chen S., Zhou Y., Chen Y., Gu J. (2018). fastp: An ultra–fast all–in–one FASTQ preprocessor. Bioinformatics.

[42] Kim D., Langmead B., Salzberg S.L. (2015). HISAT: A fast spliced aligner with low memory requirements. Nat. Methods.

[43] Pertea M., Pertea G.M., Antonescu C.M., Chang T.C., Mendell J.T., Salzberg S.L. (2015). StringTie enables improved reconstruction of a transcriptome from RNA–seq reads. Nat. Biotechnol..

[44] Pertea M., Kim D., Pertea G.M., Leek J.T., Salzberg S.L. (2016). Transcript–level expression analysis of RNA–seq experiments with HISAT, StringTie and Ballgown. Nat. Protoc..

[45] Kanehisa M., Goto S. (2000). KEGG: Kyoto encyclopedia of genes and genomes. Nucleic Acids Res..

[46] Argaw-Denboba A., Schmidt T.S.B., Di Giacomo M., Ranjan B., Devendran S., Mastrorilli E., Lloyd C.T., Pugliese D., Paribeni V., Dabin J. (2024). Paternal microbiome perturbations impact offspring fitness. Nature.

[47] Li G.Z., Zhang X.X., Li F.D., La Y.F., Zhang D.Y., Li X.L., Zhang Y.K., Song Q.Z., Zhao Y., Wang W.M. (2020). Growth and development characteristics and growth model of Hu sheep in the fattening period. Pratacultural Sci..

[48] Su L., Zhao C., Sun B., Dou L., Wang C., Yang Z., Li T., Jin Y. (2024). Effects of exercise on muscle fiber conversion, muscle development and meat quality of Sunit sheep. Meat Sci..

[49] Kong L., Yue Y., Li J., Yang B., Chen B., Liu J., Lu Z. (2023). Transcriptomics and metabolomics reveal improved performance of Hu sheep on hybridization with Southdown sheep. Food Res. Int..

[50] Ding R., Zhuang Z., Qiu Y., Ruan D., Wu J., Ye J., Cao L., Zhou S., Zheng E., Huang W. (2022). Identify known and novel candidate genes associated with backfat thickness in duroc pigs by large–scale genome–wide association analysis. J. Anim. Sci..

[51] Zhao Y., Zhang X., Li F., Zhang D., Zhang Y., Li X., Song Q., Zhou B., Zhao L., Wang J. (2022). Whole genome sequencing analysis to identify candidate genes associated with the rib eye muscle area in Hu sheep. Front. Genet..

[52] Li M., Yang R., Zhang H., Wang S., Chen D., Lin S. (2019). Development of a flavor fingerprint by HS–GC–IMS with PCA for volatile compounds of *Tricholoma matsutake* Singer. Food Chem..

[53] Li X., Wang Y., Xu J., Yang Q., Sha Y., Jiao T., Zhao S. (2024). Effects of yeast cultures on meat quality, flavor composition and rumen microbiota in lambs. Curr. Res. Food. Sci..

[54] Yang T.Y., Ma X.Y., Gong X.X., Peng C., Huang J., Zhao G.Q., Zhan K. (2022). Effects of different protein levels on gluconeogenesis of Holstein bull calves. J. China Agric. Univ..

[55] Wang Z., An X., Yang Y., Zhang L., Jiao T., Zhao S. (2023). Comprehensive analysis of the longissimus dorsi transcriptome and metabolome reveals the regulatory mechanism of different varieties of meat quality. J. Agric. Food Chem..

[56] Wang B., Sun H., Wang D., Liu H., Liu J. (2022). Constraints on the utilization of cereal straw in lactating dairy cows: A review from the perspective of systems biology. Anim. Nutr..

[57] De Vadder F., Kovatcheva–Datchary P., Goncalves D., Vinera J., Zitoun C., Duchampt A., Bäckhed F., Mithieux G. (2014). Microbiota–generated metabolites promote metabolic benefits via gut–brain neural circuits. Cell.

[58] Arshad M.S., Sohaib M., Ahmad R.S., Nadeem M.T., Imran A., Arshad M.U., Kwon J.H., Amjad Z. (2018). Ruminant meat flavor influenced by different factors with special reference to fatty acids. Lipids Health Dis..

[59] Madruga M., Dantas I., Queiroz A., Brasil L., Ishihara Y. (2013). Volatiles and water–and fat–soluble precursors of Saanen goat and cross Suffolk lamb flavour. Molecules.

[60] Salter A.M. (2013). Dietary fatty acids and cardiovascular disease. Animal.

[61] Nava Lauson C.B., Tiberti S., Corsetto P.A., Conte F., Tyagi P., Machwirth M., Ebert S., Loffreda A., Scheller L., Sheta D. (2023). Linoleic acid potentiates CD8^+^ T cell metabolic fitness and antitumor immunity. Cell Metab..

[62] Fei S., Chen Z., Xia Y., Liu H., Han D., Jin J., Xie S. (2021). Effects of dietary arachidonic acid on reproduction performance, tissue fatty acid profile and gonadal steroidogenesis in female yellow catfish pelteobagrus fulvidraco. Aquac. Nutr..

[63] Wang D., Chen L., Tang G., Yu J., Chen J., Li Z., Cao Y., Lei X., Deng L., Wu S. (2023). Multi–omics revealed the long–term effect of ruminal keystone bacteria and the microbial metabolome on lactation performance in adult dairy goats. Microbiome.

[64] Shabat S.K., Sasson G., Doron-Faigenboim A., Durman T., Yaacoby S., Berg Miller M.E., White B.A., Shterzer N., Mizrahi I. (2016). Specific microbiome–dependent mechanisms underlie the energy harvest efficiency of ruminants. ISME J..

[65] Wang K., Song D., Zhang X., Datsomor O., Jiang M., Zhao G. (2024). Effects of high–grain diet on performance, ruminal fermentation, and rumen microbial flora of lactating holstein dairy cows. Animals.

[66] Wang Z.B., Xin H.S., Bao J., Duan C.Y., Chen Y., Qu Y.L. (2015). Effects of hainanmycin or monensin supplementation on ruminal protein metabolism and populations of proteolytic bacteria in Holstein heifers. Anim. Feed Sci. Technol..

[67] Tomkins N.W., Denman S.E., Pilajun R., Wanapat M., McSweeney C.S., Elliott R. (2015). Manipulating rumen fermentation and methanogenesis using an essential oil and monensin in beef cattle fed a tropical grass hay. Anim. Feed Sci. Technol..

[68] Keogh K., Kenny D.A., Alexandre P.A., Waters S.M., McGovern E., McGee M., Reverter A. (2024). Relationship between the rumen microbiome and liver transcriptome in beef cattle divergent for feed efficiency. Anim. Microbiome.

[69] Wang H., Zhan J., Zhao S., Jiang H., Jia H., Pan Y., Zhong X., Huo J. (2024). Microbial–metabolomic exploration of tea polyphenols in the regulation of serum indicators, liver metabolism, rumen microorganisms, and metabolism in Hu sheep. Animals.

[70] Gálvez E.J.C., Iljazovic A., Amend L., Lesker T.R., Renault T., Thiemann S., Hao L., Roy U., Gronow A., Charpentier E. (2020). Distinct polysaccharide utilization determines interspecies competition between intestinal *Prevotella* spp.. Cell Host Microbe..

[71] Betancur-Murillo C.L., Aguilar-Marín S.B., Jovel J. (2022). Prevotella: A key player in ruminal metabolism. Microorganisms.

[72] Kaakoush N.O. (2015). Insights into the role of erysipelotrichaceae in the human host. Front. Cell Infect. Microbiol..

[73] Fukami T. (2015). Historical contingency in community assembly: Integrating niches, species pools, and priority effects. Annu. Rev. Ecol. Evol. Syst..

[74] Culp E.J., Goodman A.L. (2023). Cross–feeding in the gut microbiome: Ecology and mechanisms. Cell Host Microbe.

[75] Wahlström A., Brumbaugh A., Sjöland W., Olsson L., Wu H., Henricsson M., Lundqvist A., Makki K., Hazen S.L., Bergström G. (2024). Production of deoxycholic acid by low–abundant microbial species is associated with impaired glucose metabolism. Nat. Commun..

[76] Xiong L., Yao X., Pei J., Wang X., Guo S., Cao M., Bao P., Wang H., Yan P., Guo X. (2024). Do microbial–gut–muscle mediated by SCFAs, microbial–gut–brain axis mediated by insulin simultaneously regulate yak IMF deposition?. Int. J. Biol. Macromol..

[77] Jiang Y., Tang S., Wang C., Wang Y., Qin Y., Wang Y., Zhang J., Song H., Mi S., Yu F. (2018). A genome–wide association study of growth and fatness traits in two pig populations with different genetic backgrounds. J. Anim. Sci..

[78] La Y., Zhang X., Li F., Zhang D., Li C., Mo F., Wang W. (2019). Molecular characterization and expression of SPP1, LAP3 and LCORL and their association with growth traits in sheep. Genes.

[79] Soto-Pedre E., Siddiqui M.K., Mordi I., Maroteau C., Soto-Hernaez J., Palmer C.N.A., Pearson E.R., Leese G.P. (2021). Evidence of a causal relationship between serum thyroid–stimulating hormone and osteoporotic bone fractures. Eur. Thyroid. J..

[80] Wu X.F., Liu Y., Wang Y.G., Zhang F., Li W.Y. (2024). A novel 22–bp InDel within *FGF7* gene is significantly associated with growth traits in goat. Anim. Biotechnol..

[81] Pan X., Liu B., Chen S., Ding H., Yao X., Cheng Y., Xu D., Yin Y., Dai X., Sun J. (2019). Nr4a1 as a myogenic factor is upregulated in satellite cells/myoblast under proliferation and differentiation state. Biochem. Biophys. Res. Commun..

[82] Cortez-Toledo O., Schnair C., Sangngern P., Metzger D., Chao L.C. (2017). Nur77 deletion impairs muscle growth during developmental myogenesis and muscle regeneration in mice. PLoS ONE.

[83] Yilmaz O., Kizilaslan M., Arzik Y., Behrem S., Ata N., Karaca O., Elmaci C., Cemal I. (2022). Genome–wide association studies of preweaning growth and in vivo carcass composition traits in Esme sheep. J. Anim. Breed. Genet..

[84] Deng K., Fan Y., Liang Y., Cai Y., Zhang G., Deng M., Wang Z., Lu J., Shi J., Wang F. (2021). FTO–mediated demethylation of GADD45B promotes myogenesis through the activation of p38 MAPK pathway. Mol. Ther. Nucleic Acids.

[85] Matsumoto H., Kohara R., Sugi M., Usui A., Oyama K., Mannen H., Sasazaki S. (2019). The non–synonymous mutation in bovine *SPP1* gene influences carcass weight. Heliyon.

[86] Zhang S., Xu H., Liu X., Yang Q., Pan C., Lei C., Dang R., Chen H., Lan X. (2017). The muscle development transcriptome landscape of ovariectomized goat. R. Soc. Open. Sci..

[87] Smith J.A.B., Murach K.A., Dyar K.A., Zierath J.R. (2023). Exercise metabolism and adaptation in skeletal muscle. Nat. Rev. Mol. Cell Biol..

[88] Guimarães-Ferreira L. (2014). Role of the phosphocreatine system on energetic homeostasis in skeletal and cardiac muscles. Einstein.

[89] Tabe Y., Lorenzi P.L., Konopleva M. (2019). Amino acid metabolism in hematologic malignancies and the era of targeted therapy. Blood.

[90] Kindt A., Liebisch G., Clavel T., Haller D., Hörmannsperger G., Yoon H., Kolmeder D., Sigruener A., Krautbauer S., Seeliger C. (2018). The gut microbiota promotes hepatic fatty acid desaturation and elongation in mice. Nat. Commun..

