# Supplementary figures and images for: A Comparison Between High- and Low-Performing Lambs and Their Impact on the Meat Quality and Development Level Using a Multi-Omics Analysis of Rumen Microbe–Muscle–Liver Interactions
Source: Microorganisms. 2025 Apr 19;13(4):943. doi: 10.3390/microorganisms13040943 (PMC12029538; doi:10.3390/microorganisms13040943)

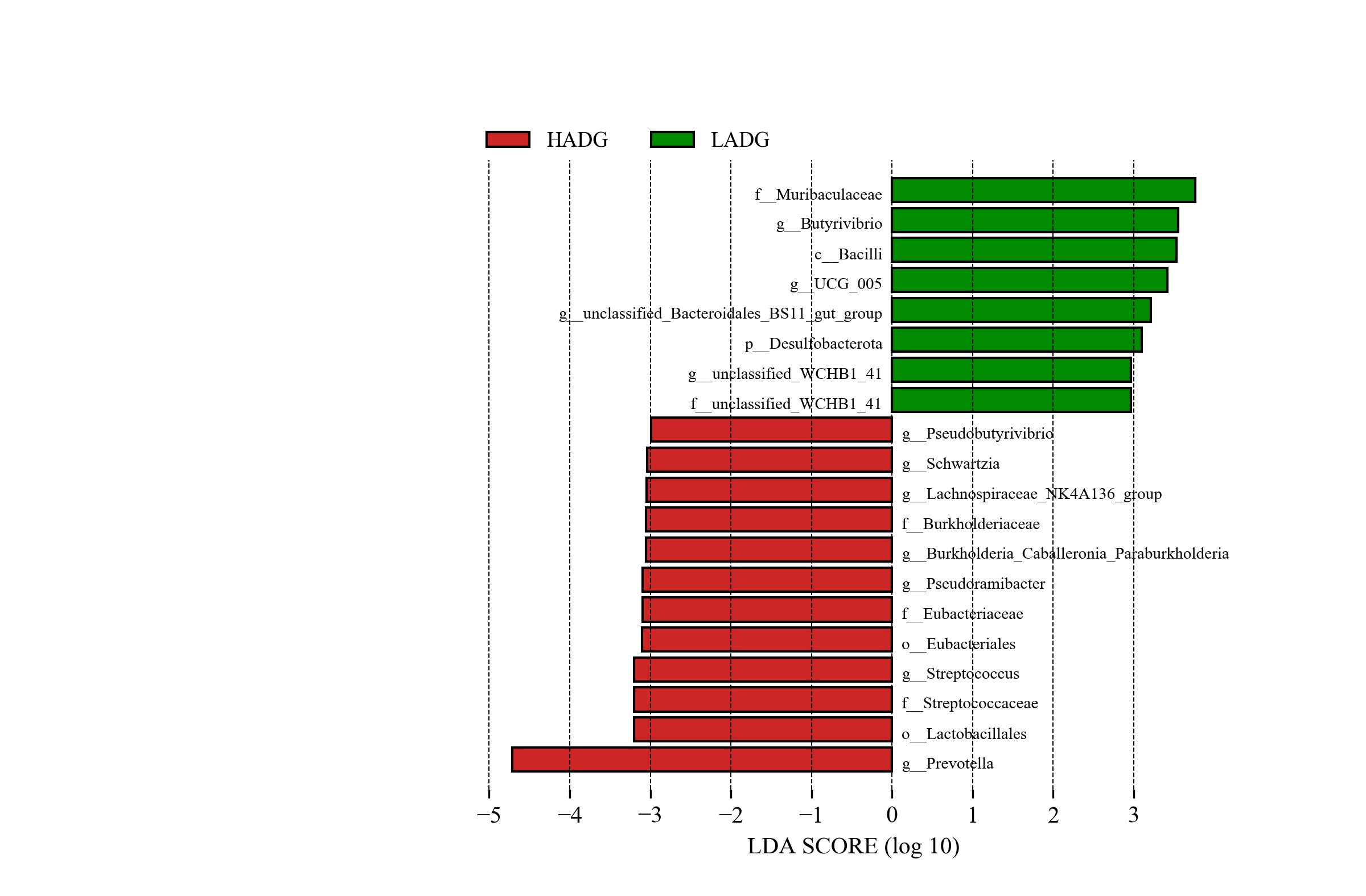

Supplement: Supplementary file 1 [file microorganisms-13-00943-s001.zip › Supplementary Figure S1 LEfSe analysis rumen biomarkers in HADG and LADG lambs.png]

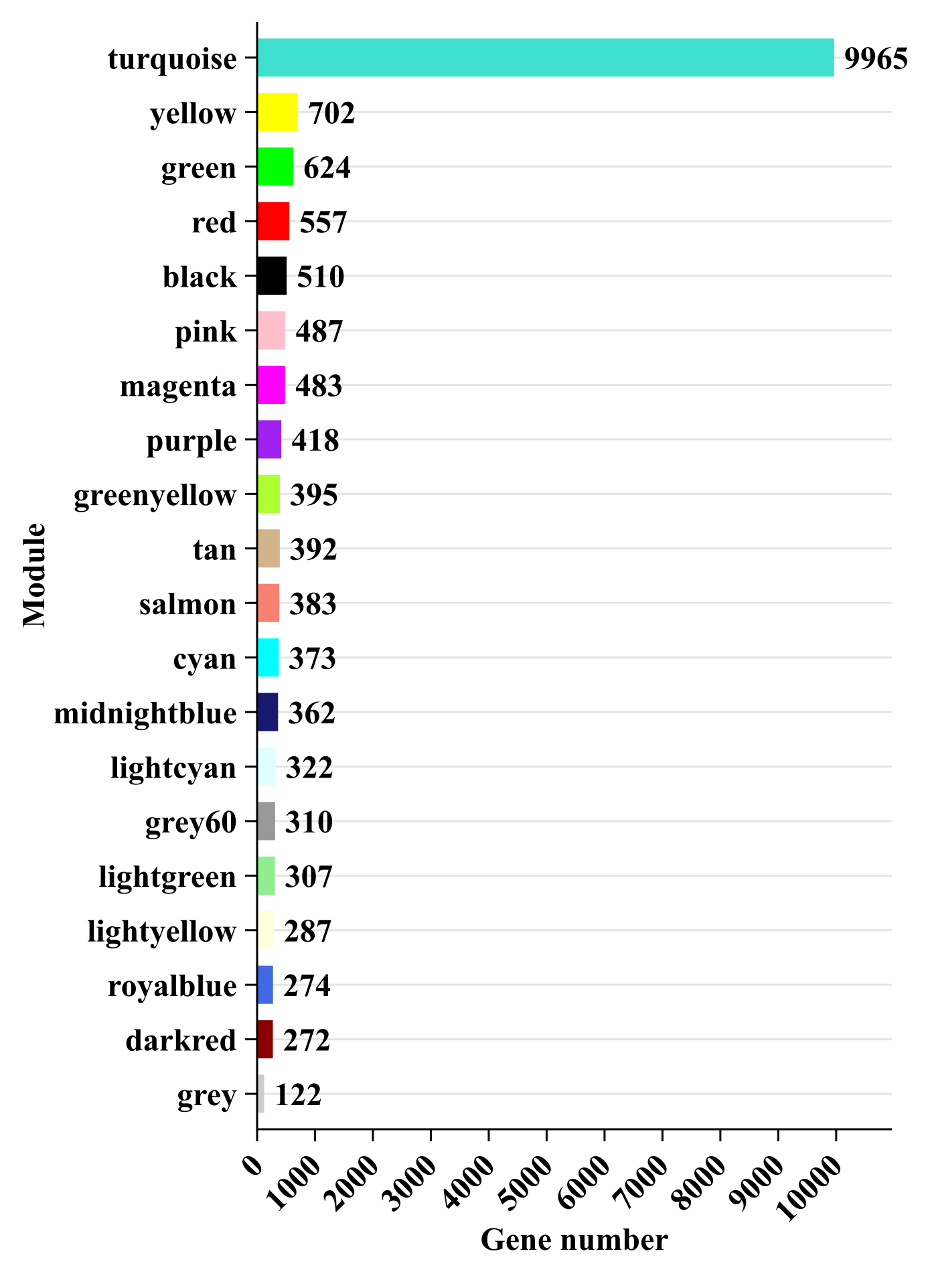

Supplement: Supplementary file 1 [file microorganisms-13-00943-s001.zip › Supplementary Figure S2 Histogram of the number of lamb longissimus dorsi genes enriched in different modules.png]

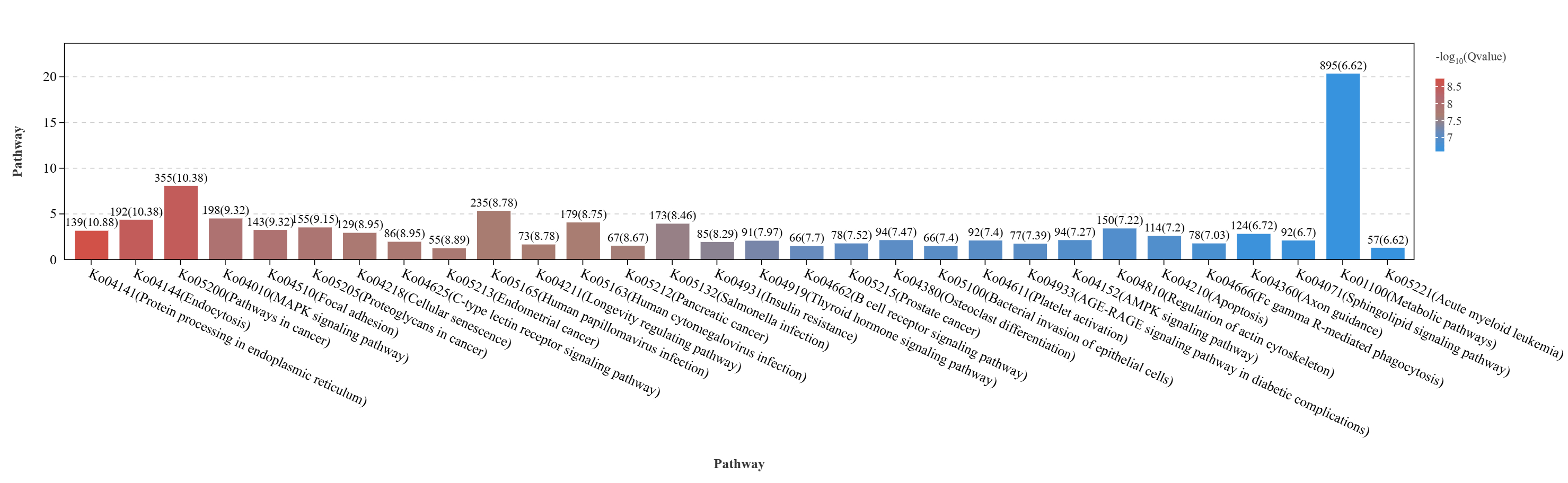

Supplement: Supplementary file 1 [file microorganisms-13-00943-s001.zip › Supplementary Figure S3 Bar graph of KEGG top 30 enrichment of turquoise module transcripts..jpeg]

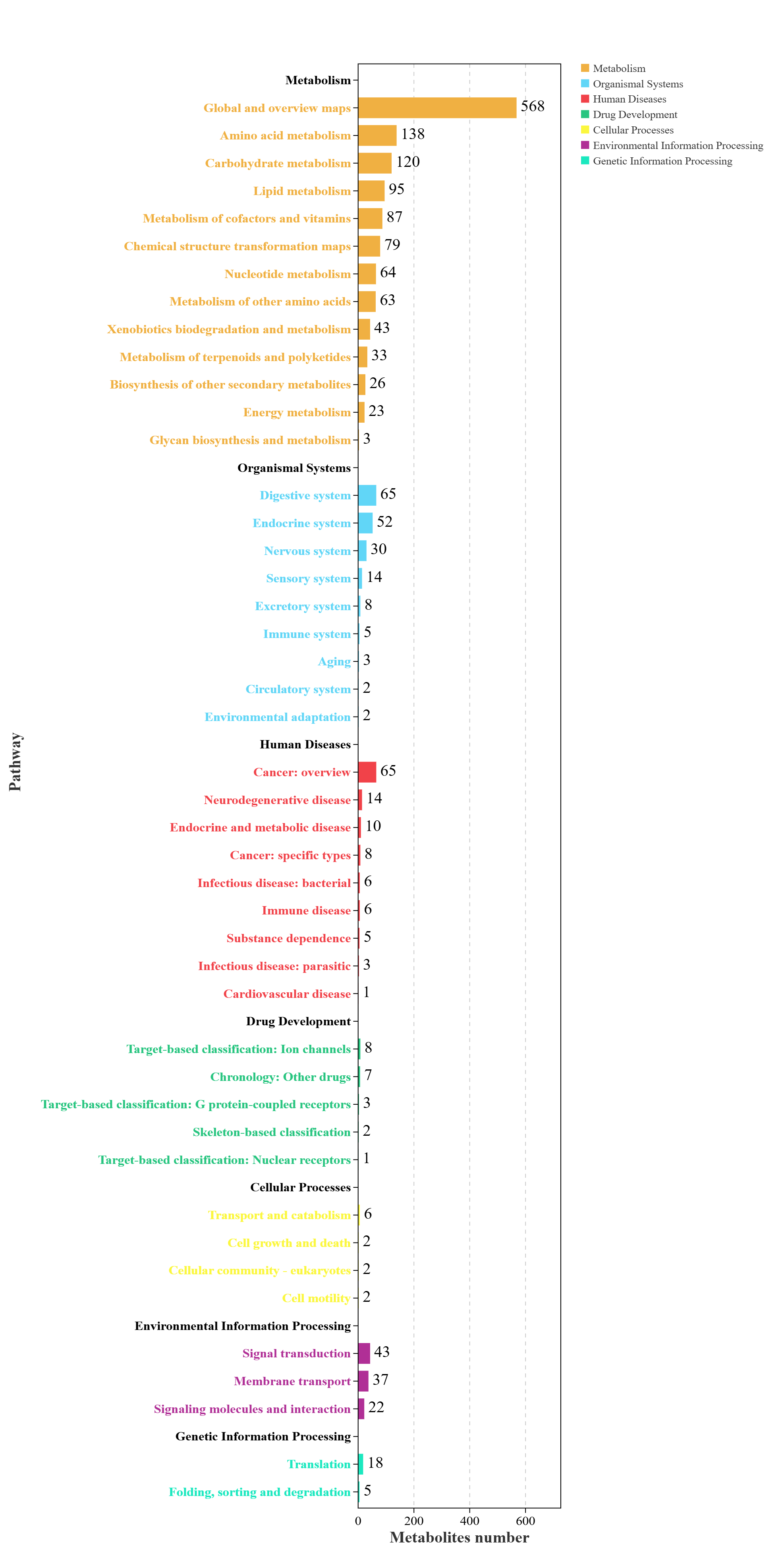

Supplement: Supplementary file 1 [file microorganisms-13-00943-s001.zip › Supplementary Figure S4 longissimus dorsi metabolite KEGG enrichment classification number histograms.jpeg]

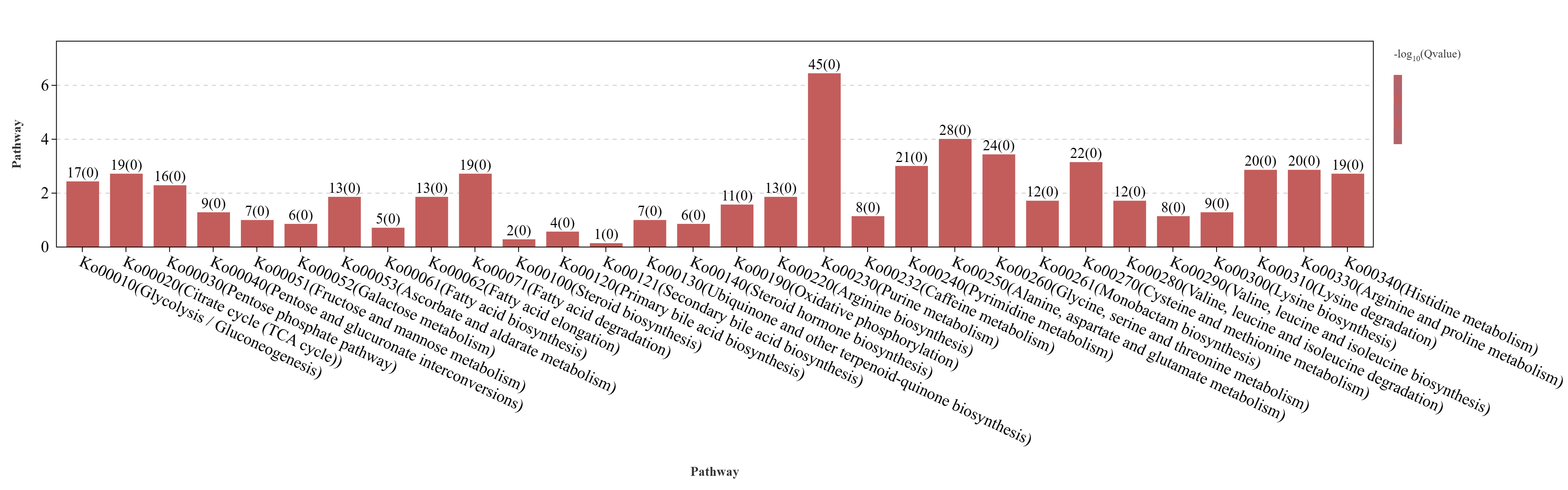

Supplement: Supplementary file 1 [file microorganisms-13-00943-s001.zip › Supplementary Figure S5 longissimus dorsi metabolite KEGG-enriched pathway FDR value top30 histogram.jpeg]

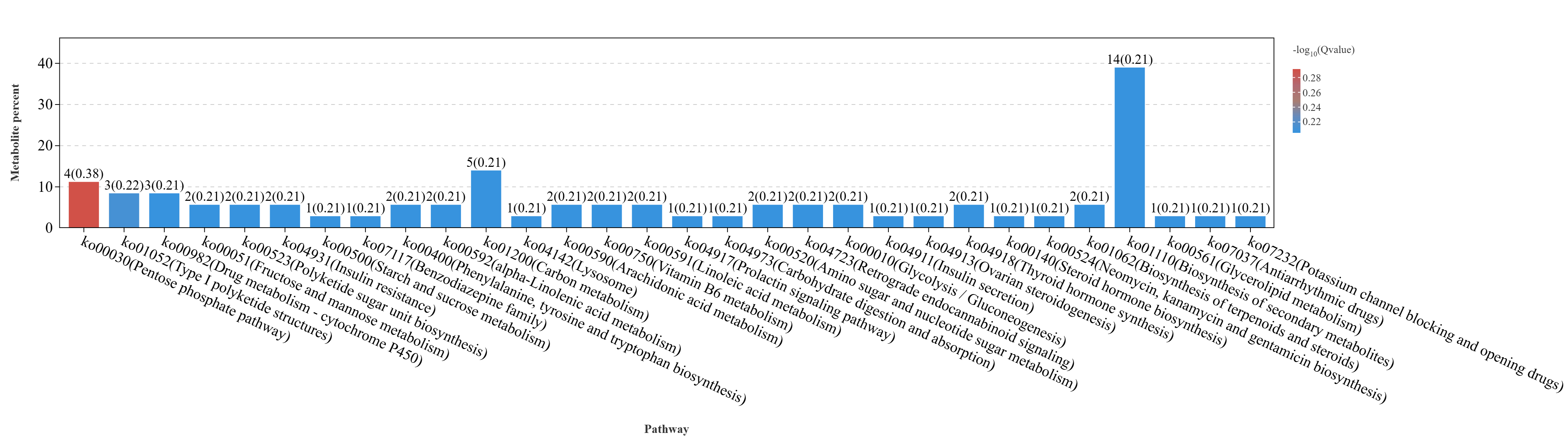

Supplement: Supplementary file 1 [file microorganisms-13-00943-s001.zip › Supplementary Figure S6 longissimus dorsi Yellow modules KEGG-enriched FDR values top30 histograms.jpeg]
